# Spatial collagen stiffening promotes collective breast cancer cell invasion by reinforcing extracellular matrix alignment

**DOI:** 10.1038/s41388-022-02258-1

**Published:** 2022-03-15

**Authors:** Thijs Koorman, Karin A. Jansen, Antoine Khalil, Peter D. Haughton, Daan Visser, Max A. K. Rätze, Wisse E. Haakma, Gabrielè Sakalauskaitè, Paul J. van Diest, Johan de Rooij, Patrick W. B. Derksen

**Affiliations:** 1 Departments of Pathology, University Medical Center Utrecht, Utrecht, The Netherlands; 2 Molecular Cancer Research/Center for Molecular Medicine, University Medical Center Utrecht, Utrecht, The Netherlands

**Keywords:** Breast cancer, Mechanisms of disease

## Abstract

The tumor micro-environment often contains stiff and irregular-bundled collagen fibers that are used by tumor cells to disseminate. It is still unclear how and to what extent, extracellular matrix (ECM) stiffness *versus* ECM bundle size and alignment dictate cancer cell invasion. Here, we have uncoupled Collagen-I bundling from stiffness by introducing inter-collagen crosslinks, combined with temperature induced aggregation of collagen bundling. Using organotypic models from mouse invasive ductal and invasive lobular breast cancers, we show that increased collagen bundling in 3D induces a generic increase in breast cancer invasion that is independent of migration mode. However, systemic collagen stiffening using advanced glycation end product (AGE) crosslinking prevents collective invasion, while leaving single cell invasion unaffected. Collective invasion into collagen matrices by ductal breast cancer cells depends on Lysyl oxidase-like 3 (Loxl3), a factor produced by tumor cells that reinforces local collagen stiffness. Finally, we present clinical evidence that collectively invading cancer cells at the invasive front of ductal breast carcinoma upregulate LOXL3. By uncoupling the mechanical, chemical, and structural cues that control invasion of breast cancer in three dimensions, our data reveal that spatial control over stiffness and bundling underlie collective dissemination of ductal-type breast cancers.

## Introduction

Breast cancer invasion strongly depends on deposition and modification of the extracellular matrix (ECM) [1]. Tumors often show desmoplasia or abundant ECM deposition, including Collagen-I and ECM modifiers that render cancerous tissues up to ten times stiffer and denser compared to their healthy counterparts [2]. Indeed, tissue stiffness has been correlated with cancer progression, where stiffness increased with cancer grade [3, 4]. Interestingly, the presence of thick aligned collagen fibers at the invasive front of a primary tumor is correlated to worse patient outcome in breast and other cancers [5, 6]. Based on these findings, it has been proposed that cancer cells use these highly aligned collagen structures as ‘highways’ for invasion and dissemination [7]. However, it remains unclear whether the structural composition of the aligned collagen bundles or the stiffness of the bundles is the rate-limiting factor. Efforts to uncouple the effect of bundling *versus* stiffness in the context of 3D cancer cell migration have mostly varied both collagen concentration and network pore size, which precludes uncoupling of the two ECM variables [8, 9]. Interestingly, evidence using Advanced Glycation End (AGE) product-induced collagen crosslinking indicated that invasion of intestinal adenocarcinoma cells could be inhibited if network stiffening was induced independently from the network structure [10]. These results contradict data from primary human samples, where stiffness of the breast is a clear risk factor for the development of breast cancer [11], and increased stiffness is correlated with increased invasiveness and tumor grade [3, 12].

Histological classification of breast cancer is largely based on cellular and nuclear morphology and mode of invasion, which results in two main types: invasive ductal breast cancer of no special type (IDC-NST) and invasive lobular breast cancer (ILC). IDC comprises approximately ~80% of all breast cancer cases and is characterized by collective invasion, whereas ILC typically invades and metastasizes as single cells and single layer strands [13]. Collective invasion modes in breast cancer have been linked to an acquisition of myoepithelial or ‘basal’ characteristics, whereby luminal-type breast cancers can acquire expression of markers such as cytokeratin (CK) 5, CK14 and p63 in invading leader cells that are in contact with the tumor stroma [14]. However, despite strong evidence that expression of basal markers is necessary for collective invasion [14, 15], most human breast cancers are luminal in nature, with only 10–15% diagnosed as the basal subtype with spindle cell phenotypes [16]. Therefore, acquisition of basal invasive characteristics may be spatiotemporally regulated and subject to local modulation of the ECM viscoelasticity [17]

Stiffness of the tumor micro-environment can be controlled by collagen crosslinkers such as the Lysyl oxidases, which consist of Lysyl oxidase (LOX) and the Lox-like 1–4 (LOXL1–4) proteins; amine oxidases that share high similarity in their copper-dependent catalytic domain [18]. All LOX family members cross-link collagens and Elastin [18], and LOX-dependent crosslinking has been linked to increased metastasis and cancer growth [19, 20]. In breast cancer, inhibition of the crosslinking activity of the LOX family with βAPN prevents metastasis in an IDC-type mouse model [21, 22], showing that LOX proteins promote cancer metastasis by enhanced ECM crosslinking and subsequent stiffness.

To better understand the independent contribution of structure and stiffness during cancer invasion, we studied the interplay of breast cancer invasion and the ECM by uncoupling collagen bundling from stiffness. For this, we used mouse organotypic models of IDC or ILC and show that during collective invasion of IDC, the collagen matrix is aligned towards the cancer organoid by basal-like leader cells. Interestingly, our data show that collective cancer invasion is inhibited upon systemic and homogeneous collagen stiffening, independent of the collagen structure, whereas single cell invasion is unaffected. Our data suggest that Loxl3 expression by basal leader cells induces local ECM stiffening, thereby enabling collective invasion of ductal-type breast cancer.

## Results

### Breast cancer invasion is accompanied by remodeling of the extracellular matrix

**Fig. 1:**
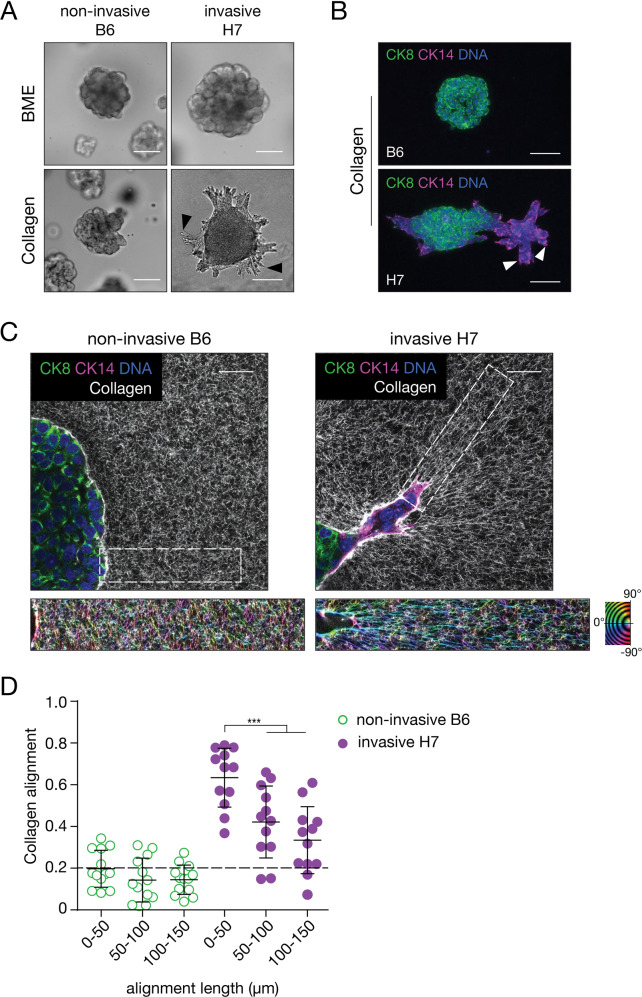
Ductal-type mouse breast cancer organoid models acquire basal features and align Collagen-I rich environments during collective invasion. **A** Non-invasive tumor PyMT organoid clones B6 (left panels) or invasive clones H7 (right panels) were placed in BME (top panels) or Collagen-I (bottom panels). Note the overt invasion of H7 in collagen (black arrow heads) in contrast to the non-invasive clone B6. Size bars indicate 50 µm. **B** Invasion is accompanied by expression of basal cytokeratin (CK) expression. Invasive PyMT H7 organoids show expression of CK14. In contrast, the non-invasive clone B6 does not acquire CK14 expression but instead shows homogenous CK8 expression. White arrow heads depict the basal-type leader cells. Size bars indicate 75 µm. **C**, **D** Invasive organoids align the collagen network. Collagen alignment (collagen bundle angle variability perpendicular to the organoid) by collectively invading H7 organoids was visualized using confocal reflection microscopy (insets) (**C**) and quantified using OrientationJ in (**D**). Pseudo coloring in the insets indicate orientation angles from −90° to 90°, perfect alignment with the organoids is defined as 1, and the cut-off for non (random)-alignment is 0.2 in (**D**). Alignment persisted over long distances (>150 µm). In contrast, non-invasive PyMT B6 organoids did not induce collagen matrix alignment. Size bar indicates 10 µm. The Mann–Whitney test was done to assess significance. **p < 0.01, ***p < 0.005, ****p < 0.001. All experiments are biological replicates and were repeated at least three times. Error bars show standard error of the mean.

To study breast cancer invasion into the surrounding collagen-rich matrix, we derived primary organotypic tumor cultures from invasive mammary ductal-type carcinomas that had developed in MMTV-PyMT mice [23]. We next selected a non-invasive clone (B6) and an invasive PyMT clone (H7) using clonal selection based on their ability to invade a Collagen-I matrix (Fig. 1A). Interestingly, non-invasive PyMT organoids are characterized by pure luminal-type cytokeratin (CK)8-expressing (CK8^HIGH^) cells, while the invasive organoids also contained CK14 expressing basal CK14^HIGH^CK8^LOW^ cells at the organoid periphery (Fig. 1B). In line with published results [14], we observe that collective invasion of the PyMT mouse-derived tumor organoid (MDO) models in Collagen-I is restricted to the peripheral basal-type invading cells (Fig. 1B). Neither the non-invasive B6 nor the invasive H7 MDO show invasion in a Laminin/Collagen-IV rich basement membrane extract (BME) (Fig. 1A). Invasion into the surrounding matrix is accompanied by a spatial loss of junctional E-cadherin expression (Supplementary Fig. 1) and alignment of the surrounding collagen matrix (Fig. 1C,D). Collagen alignment was quantified by selecting regions of interest within the ECM that are positioned perpendicular to the cell-ECM border (Fig. 1C). Experimentally, we considered values for collagen alignment below 0.2 as isotropic (dotted line). Collagen alignment is highest close to the organoid-ECM border (0–50 µm) for the invasive PyMT H7 model, while collagen is not aligned for the non-invasive B6 PyMT-MDO (Fig. 1D). Notably, collagen alignment persists over relatively large distances from the invasive organoid (>100 μm). This long-range alignment due to cellular forces has previously been attributed to the fibrous nature of the ECM [24, 25] and is in line with previous observations [26].

**Fig. 2:**
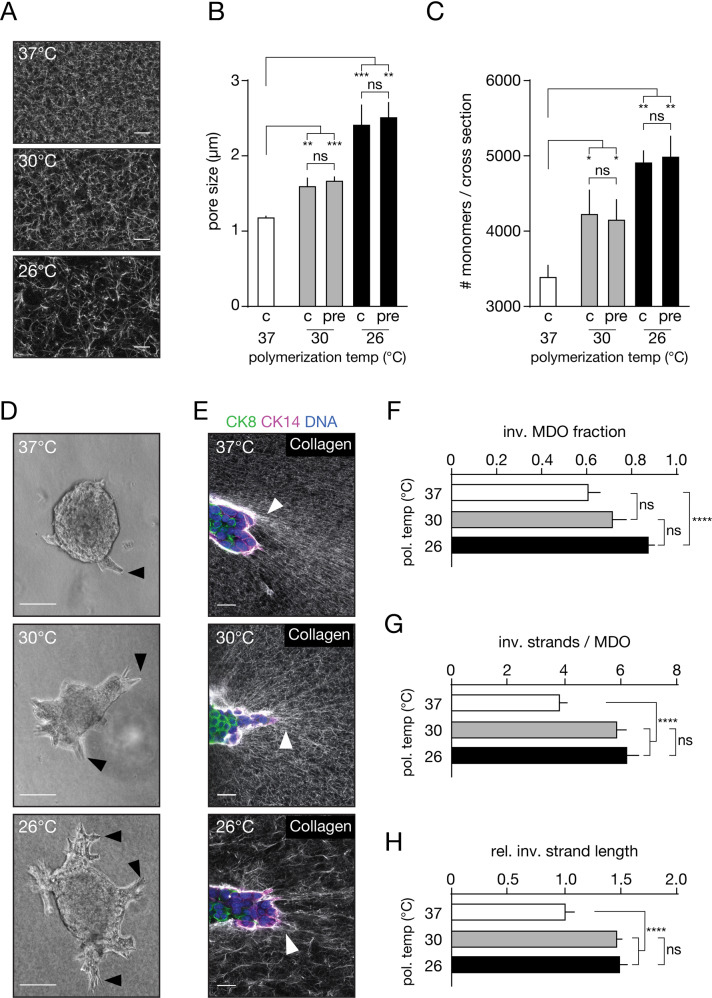
Increasing collagen fiber thickness increases breast cancer invasion. **A** Collagen structure can be modulated by (pre-)polymerizing matrices at the indicated temperatures. Size bar indicates 10 µm. **B** Quantification of the pore size of confocal images presented in (**A**). Collagen structures were set when pre-polymerizing (pre) for approximately 45 min at the indicated temperatures and did not significantly differ compared to non-varying constant (c) temperatures. **C** Collagen monomer numbers in a collagen fiber increase with decreasing (pre)-polymerization temperatures. We obtained no differences between pre-polymerizing for a minimum of 45 min. (pre), or polymerizing for 3 h (c) at the indicated temperatures. **D** Collagen bundling increases PyMT MDO invasion. Black arrow heads indicate the leader cells of the invasive strands. Size bar indicates 75 µm. **E** Confocal images of collagen-embedded organoids under varying bundling conditions. Invasion was accompanied by aligning of the collagen matrix towards the invasive leader cells (white arrow heads). Size bar indicates 20 µm. **F**–**H** Quantification of (**D**), where the fraction of invasive organoids are depicted. **F**, the number of invasive strands per organoid (**G**), and the relative length of those strands compared to the 37 ^o^C condition (**H**). Experiments are biological replicates and were repeated at least three times. More than 50 organoid structures were quantified per experiment. Statistical significance was assessed by the Kruskal–Wallis test. Error bars show standard error of the mean. *p < 0.05; **p < 0.01; ***p < 0.005; ****p < 0.001.

### Thick bundles of Collagen-I fibers promote cancer invasion

To define which physical parameters determine ECM-driven breast cancer invasiveness, we induced structural collagen bundling by varying the polymerization temperature of an experimental 3D Collagen-I ECM. By pre-polymerizing at either 26 °C or 30 °C (1 h), we generated matrices with increased fiber diameters compared to the 37 °C control (Fig. 2A). Decreasing the pre-polymerization temperature increases the pore size from approximately 1.2 µm when polymerized at 37 °C, to 1.6 µm and 2.6 µm when pre-polymerized at 30 °C or 26 °C, respectively (Fig. 2B). As expected, larger pore sizes are accompanied by an increase in the number of collagen monomers inside a collagen fiber as measured by turbidity assays (Fig. 2C), confirming an established correlation between collagen pore size, fiber thickness, and polymerization temperature [27]. In agreement with others [28, 29], we show that decreasing the pre-polymerization temperature to 26 °C markedly stiffens the collagen network compared to the 37 °C control by a factor of 3 (Supplementary Fig. 2). Since the tumor micro-environment tends to stiffen compared to its healthy counterpart and is accompanied by the formation of thick collagen bundles [3, 5], we anticipated that cancer invasion in networks polymerized at 26 °C would promote tumor cell invasiveness compared to the 37 °C counterpart. Indeed, invasion of the PyMT H7 organoid model is markedly increased with elevated collagen bundling and larger pore sizes (Fig. 2D–E). The number of collectively invading strands per organoid (Fig. 2G), invasive strand length (Fig. 2H) and relative number (fraction) of organoids that are invasive (Fig. 2F), all increase with augmented collagen bundling. These results show that increased collagen bundling, collagen stiffness, and the resulting increases in pore size significantly enhance 3D breast cancer organoid invasion.

### Uncoupling collagen stiffness from collagen structure

**Fig. 3:**
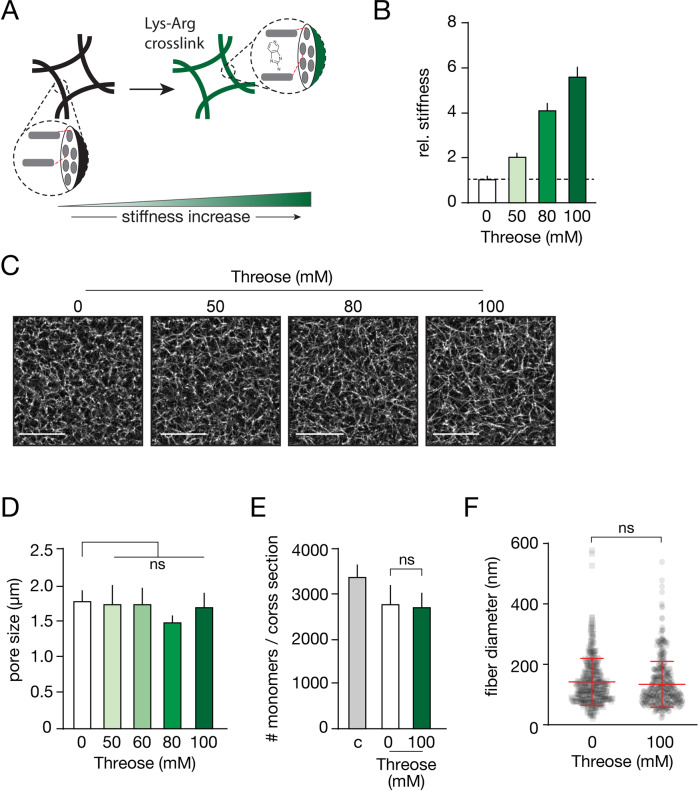
A collagen stiffening protocol that does not interfere with collagen structure. **A** Cartoon depicting the introduction of Advanced Glycation End (AGE)-crosslinkers inside the collagen fibers without changing collagen structure. **B** Introducing crosslinks by pre-incubating with the indicated threose concentrations before network formation increases collagen stiffness in a dose-dependent manner. Data were normalized to control situation (no threose). **C**, **D** Confocal microscopy images showing collagen networks formed from threose-stiffened collagen indicate that pore sizes remain unaffected. Size bar indicates 20 µm. Quantifications from (**C**) are shown in (**D**). ns Non significant. **E** Turbidity assays were performed to assess the effect of AGE crosslinking on the number of collagen monomers per fiber. Control conditions (c) were prepared directly from the collagen stocks, while the threose-free condition (0 mM) was pre-incubated with solvent only. **F** Fiber diameters from (**E**) were quantified using TEM and ImageJ. All experiments are biological replicates and were repeated at least three times. Error bars show standard error of the mean.

Thick collagen bundles enhance cancer cell invasion (Fig. 2). However, decreasing the pre-polymerization temperature also increases the stiffness and the pore sizes of the collagen network itself (Supplementary Fig. 3). It, therefore, remained unclear if collagen stiffness or collagen fiber diameter (which we designate as structure) is the rate-limiting factor for 3D collective cancer cell invasion. To examine this, we decoupled collagen structure from collagen bundle stiffness by introducing advanced glycation end product (AGE) intra-stand collagen crosslinks, which induces an increase in bundle stiffening without affecting collagen bundling [30]. AGE crosslinks were introduced by Threose (Fig. 3A), which results in stiffening of the collagen networks up to 2 to 6-fold (Fig. 3B, and Supplementary Fig. 3B), while not affecting the pore size of the network (Fig. 3C, D, and Supplementary Fig. 3C). Subsequent turbidity assays show that the number of collagen monomers in a fiber remain unaffected (Fig. 3E), which was verified by fiber diameter measurements using transmission electron microscopy (Fig. 3F). Because collagen fibers appear more linear when glycated (see Fig. 3C), it suggests that the persistence length (or bending stiffness) of these fibers increases compared to the non-glycated controls. In short, we have successfully set up a model system to stiffen Collagen-I by introducing AGE crosslinks inside collagen fibers without changing the network structure.

### Stiffer collagen matrices inhibit collective invasion

**Fig. 4:**
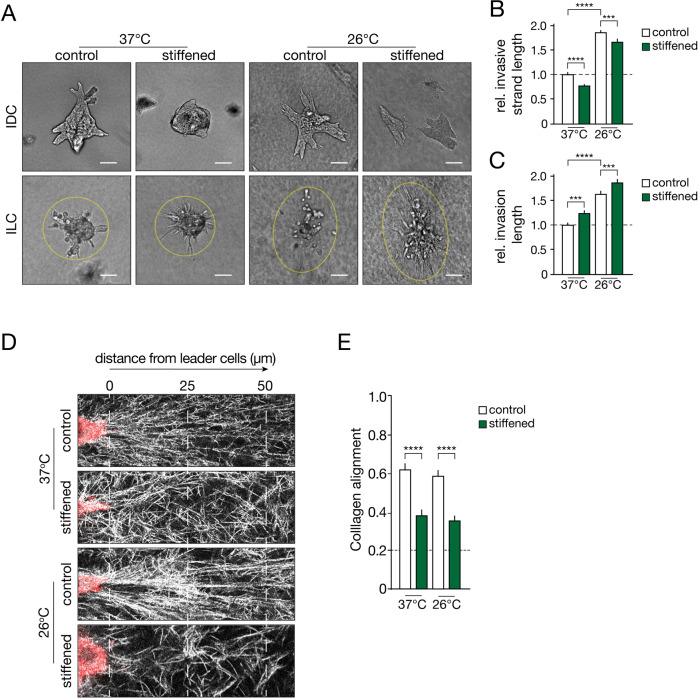
Collagen network stiffening reduces collective invasion but promotes single cell dissemination. **A** IDC (top panels) or ILC MDO cells (bottom panels) were seeded in collagen matrices polymerized at 37 °C or pre-polymerized at 26 °C, and network stiffening was induced using AGE crosslinkers (stiffened). Scale bar denotes 100 µm. **B**, **C** Quantification of the invading strands of the IDC PyMT MDO (cultured for 3 days). **B** Quantification of the length of invading strands of the IDC PyMT MDO (cultured for 3 days). **C** Quantification of length of invasion of the ILC MDO as defined by major axis of ellipse. Invasive lengths in **B** and **C** were normalized to the 37 °C control condition (dashed line). Invasive strand length was normalized to the 37 °C control conditions (dashed line). **D** Confocal images were obtained from IDC MDO model in Collagen matrices, polymerized at 37 °C or pre-polymerized at 26 °C and stiffened using AGE crosslinkers. Organoids were stained for F-actin (red) and Collagen (white). **E** Collagen alignment in (**D**) was quantified in the region 0–50 µm from the invasive leader cells after 3 days. Collagen was considered aligned at a 0.2 cut-off (dashed line). Statistical significance was assessed by the Mann–Whitney test. Error bars show standard error of the mean. ***p < 0.005. All experiments are biological replicates and were replicated at least three times.

Next, we used AGE crosslinking to investigate if collagen fiber stiffness modulates invasion independent of network structure. Interestingly, systemic stiffening of the collagen matrix reduces collective invasion of the PyMT MDO cells, irrespective of fiber thickness or pore size (Fig. 4A top panels and 4B). To study if systemic collagen stiffening also decreases single cell invasion, we generated a tumor organoid model from a conditional mouse model for human ILC (*WAPcre;Cdh1*^*F*^*;Trp53*^*F*^) [31] and assessed the effect on single cell invasion. In this model, single cell invasion is increased by collagen stiffness, independent of bundling (Fig. 4A bottom panels, and Fig. 4C). In addition, ILC cells show more invasion if matrices are stiffer and contain increased collagen bundles (Fig. 4A compare top IDC to bottom ILC panels). Of note, while stiffer environments increase the length of the invasive strands, they also induce a more spindle-shaped morphology, which is uncharacteristic of ILC. Classical ILC cells usually invade their micro-environment as rounded cells in trabecular structures [32], a feature that is apparent in the non-stiffened conditions (Supplementary Fig. 4; red arrows). As such, we find that collective invasion is inhibited by increased stiffness irrespective of collagen bundle thickness, while single cell invasion is promoted with both increased stiffness and collagen bundle size. Threose-induced crosslinking reduces collagen alignment up to approximately two-fold in both the 37 °C and 26 °C polymerization conditions (Fig. 4D, E). These findings suggest that both spatial modulation of collagen stiffness and collagen bundling/alignment over a considerable distance (at least 25–50 µm; Fig. 4D) are required to propagate the mechanical cues that underlie collective cancer cell invasion.

Our Threose-based AGE crosslinking experiments show that collagen stiffening limits collective cell invasion independently of the bundling thickness (Fig. 4). However, introducing AGE crosslinks can stimulate AGE/RAGE signaling (reviewed in: [33]), which might confound results. To ensure that the reduction of collective invasion is specifically due to stiffness, we also used a light-inducible protocol to stiffen collagen networks independently of collagen structure using Ruthenium (Ru), visible light and sodium persulfate (Na_2_S_2_O_8_) as electron donor (Supplementary Fig. 5A). Ru-dependent crosslinking induces crosslinks without affecting viability or causing DNA damage under these conditions (Supplementary Fig. 5B–E). Collective invasion of the PyMT H7 MDO is reduced upon light-induced network stiffening, independent of the network structure (Supplementary Fig. 5F, G), confirming that systemic collagen network stiffening reduces collective invasion.

In short, our data show that collective cancer cell invasion is enhanced by increased collagen bundling and alignment but inhibited by an increase in homogeneous, systemic matrix stiffness. Conversely, we find that single cell invasion does not require extensive alignment of collagen but is mainly propelled by collagen bundling and a concomitant induction of matrix stiffness.

### Expression of Loxl3 in leader cells promotes collective breast cancer cell invasion

**Fig. 5:**
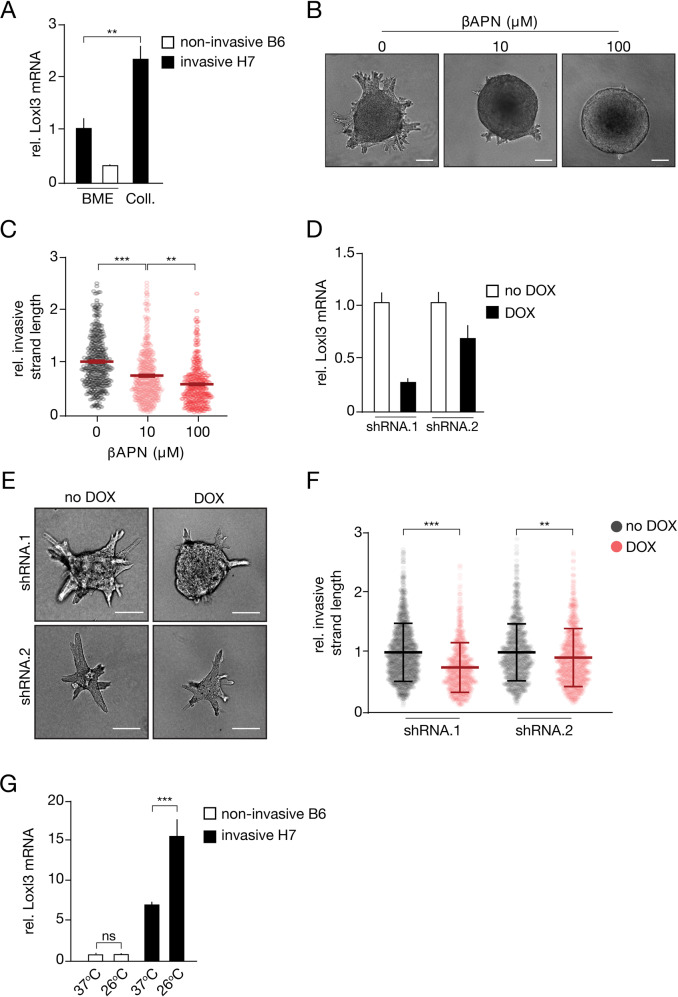
Lysyl oxidase-like 3 (Loxl3) modulates local collagen stiffness to promote collective invasion. **A** Loxl3 upregulation during invasion in collagen was validated using RT-qPCR. Invasive H7 MDO (black bars) and non-invasive B6 MDO clones (white bar) were compared in BME to depict differential Loxl3 expression in the invasive clone. Note the increase in Loxl3 expression upon transfer of the invasive H7 MDO to a collagen (Coll.) matrix. **B**, **C** Collective invasion by PyMT H7 MDO in collagen requires Lysyl oxidases. βAPN, a generic Lox family inhibitor reduces collective invasion in a dose-dependent manner. Size bar represents 100 µm. Invasive strand length was quantified and normalized to the 0 µM control in (**C**). **D** Loxl3 inducible knockdown efficiency in PyMT MDO cells. Two independent shRNA sequences were expressed as doxycyclin (DOX)-inducible constructs using viral transduction. Loxl3 mRNA expression was determined after DOX addition using RT-qPCR. Error bars show standard deviation. **E**, **F** Loxl3 downregulation attenuates collective invasion of breast cancer organoids in collagen. Shown are DIC images of PyMT-H7 MDO cells cultured in collagen matrices, pre-polymerized at 37 °C in the absence and presence of DOX. Size bar represents 100 µm. Invading strand length was quantified in (**F**) and normalized to 1 based on the no dox control. **G** Collagen network stiffening induces upregulation of Loxl3. Shown are RT-qPCR results for Loxl3 mRNA of non-invasive B6 and invasive H7 organoids grown in collagen matrices at the indicated (pre) polymerization temperatures. Error bars show standard deviation. Statistical significance was assessed by the Student’s *t*-test **p < 0.01; ***p < 0.005. All experiments were replicated at least 3 times. Statistical significance was assessed by the Mann–Whitney test (for A + F) and the Kruskal–Wallis test (for **C**). Error bars show standard error of the mean.

Because invasion of the PyMT MDO cells is propelled by collagen bundling, but attenuated when collagen bundles are systemically crosslinked, we reasoned that collectively invading cancer cells need to spatially stiffen collagen bundles. We, therefore, performed single cell mRNA sequencing and identified the genes that are upregulated in collagen when compared to BME, specifically in the CK14^HIGH^CK8^LOW^ basal cells. To identify the basal leader cells that are in contact with ECM, we classified populations based on expression of the myoepithelial markers (CK14/CK5) and downregulation of the luminal markers (CK8/CD14). From the resulting gene list, we selected the genes that can modify the ECM structure and/or function and fall within the ‘Matrisome’ (Supplementary Fig. 6A) [34]. Within this gene set, we identify several ECM glycoproteins and ECM regulators, from which we prioritized Lysyl oxidase-like 3 (LoxL3), a secreted ECM modifier implicated in collagen crosslinking and Integrin-mediated invasion processes [35, 36]. We confirm that Loxl3 mRNA expression is higher in the invasive clone H7 compared to the non-invasive clone B6 and show a transcriptional upregulation of Loxl3 when H7 organoids are cultured in collagen-rich matrices (Fig. 5A). We next generated non-invasive PyMT B6 organoids that stably express Loxl3 and observed that Loxl3 overexpression is not sufficient to drive invasion in this cell type (Supplementary Fig. 6B–E).

Because Loxl3 mRNA is transcriptionally upregulated in the invasive PyMT H7 organoids, we analyzed a region upstream of the human and mouse Loxl3 genes, and identified several TEAD consensus-binding sites up to approximately 1kB upstream of the transcription start site (Supplementary Fig. 6F, G). We focused on TEAD binding sites, because of the importance of a balanced progression of mechanical forces during collective invasion, and the known association of the TEAD family of transcription factors with the co-activator YAP. Because nuclear YAP is linked to breast cancer invasion and its function is intricately connected to mechanical responses [37–40], we analyzed its nuclear expression in the context of increased collagen stiffness and bundling during cancer cell invasion. First, nuclear YAP is enriched in the H7 MDO cells that are in contact with collagen, while the non-invasive B6 MDO model shows lower YAP nuclear/cytosolic ratios in cells that are in contact with the ECM (Supplementary Fig. 7A, B). Moreover, while collectively invading organoids show increased YAP nuclear translocation when increasing collagen bundling at 26 °C, they do not show a further increase of nuclear YAP when stiffening collagen using AGE crosslinkers (Supplementary Fig. 7C, D). Conversely, single cell invading ILC organoids show a more heterogenous pattern of nuclear YAP localization and display increased levels of nuclear YAP in AGE-crosslinked collagen, independent of collagen bundling (Supplementary Fig. 7E, F). These data indicate that localized collagen crosslinking promotes spatial increases in cellular mechanical forces to promote collective cancer cell invasion.

We next confirmed previous findings that Lysyl oxidases promote 3D cancer invasion [41] by functionally inhibiting all Lysyl oxidases. Inhibition of Lox-dependent collagen crosslinking by using β-aminopropionitrile (βAPN) reduces collective invasion in collagen in a dose-dependent manner in the invasive PyMT H7 organoid model (Fig. 5B, C), but not in the ILC organoids (Supplementary Fig. 8), suggesting that Lysyl oxidases specifically promote collective invasion in these models. This is further substantiated by an inducible knockdown of Loxl3 using two different shRNAs (Fig. 5D), which leads to a decrease in the length of the invading PyMT MDO strands (Fig. 5E, F). Finally, we observe that increasing the temperature dependent stiffness of Collagen-I leads to an upregulation of Loxl3 expression, specifically in the invasive H7 PyMT model (Fig. 5G). Based on these findings, we conclude that collective invasion of ductal-type breast cancer in Collagen-I-dense matrices can be promoted by Loxl3.

### LOXL3 expression in high-grade invasive breast cancer

**Fig. 6:**
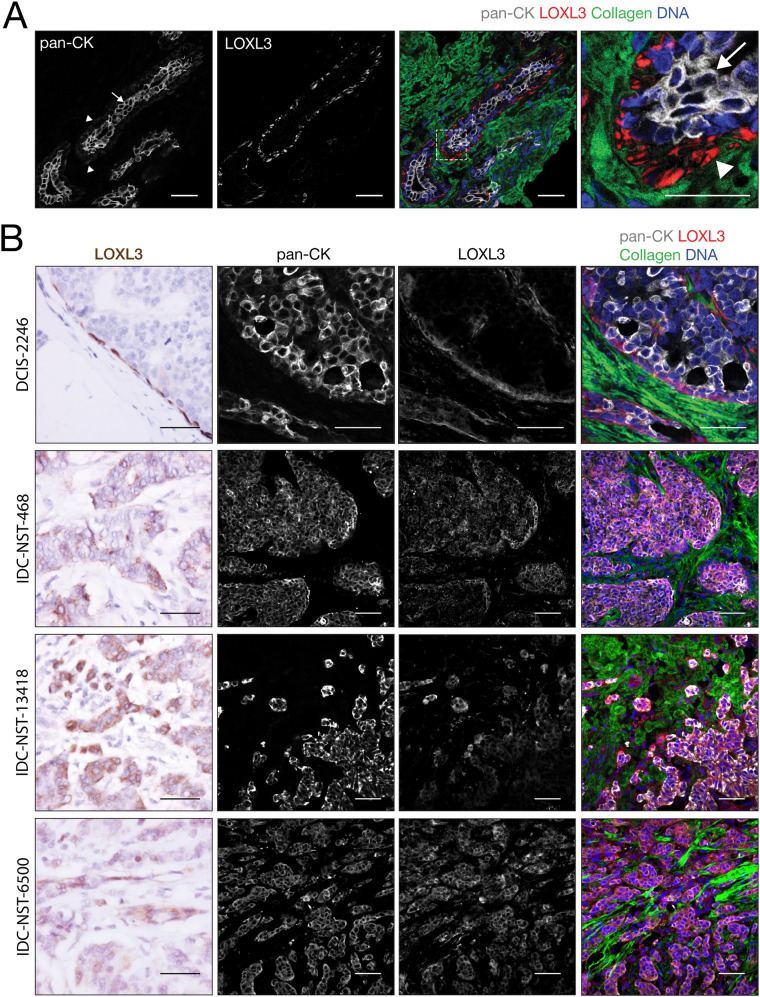
LOXL3 expression in normal mammary ducts and invasive ductal breast cancer. **A** LOXL3 is expressed by the myoepithelial cells in healthy mammary ducts. Shown is a representative confocal image (immunofluorescence) of healthy mammary ducts, showing luminal epithelial cells (arrow) and myoepithelial cells in white (left panel, arrow heads, pan-cytokeratin) and LOXL3 (red) in the context of collagen (green). Right panel depicts enlargement of the inset. Size bars represent 50 µm. Note the specific LOXL3 expression in the myoepithelial cells of the mammary gland ducts (arrow heads). **B** LOXL3 expression is upregulated at the invasive front of high grade collectively invading ductal breast cancer. Shown are LOXL3 immunohistochemistry (IHC; brown) and immunofluorescence (IF) examples of a ductal carcinoma in situ (DCIS) and high-grade IDC-NST samples. IF samples were co-stained for pan-cytokeratins (pan-CK; white), collagen (green) and (DNA; blue). Middle panels show the greyscale images corresponding to the merged signals in the right panels. Note that LOXL3 expression is confined to the myoepithelial cells in the DCIS sample, whereas IDC/NST samples express LOXL3 in the invasive tumor cells. Size bars represent 50 µm.

Given the pro-invasive function of Loxl3 in the PyMT MDO model, we investigated LOXL3 protein expression in human breast tissue and primary IDC-NST samples. Using immunofluorescence, we observe that LOXL3 is exclusively expressed by the myoepithelial cells of the healthy mammary duct (Fig. 6A). Next, we employed conventional immunohistochemistry (IHC) to analyze LOXL3 expression in IDC-NST. For this, we first used a selection of whole slide samples to delineate spatial expression patterns. The whole slides also contain normal mammary epithelium that serves as a specificity control for LOXL3 expression. In addition, we observe that LOXL3 is expressed by the circumferent myoepithelial cells in ductal carcinoma in situ (DCIS), but not by the carcinoma cells (Fig. 6B, top panels). In IDC- NST, LOXL3 is expressed and enriched in focal areas at the invasive front by tumor cells that are in direct contact with the stroma (Fig. 6B). We detect LOXL3 expression in 5 out of the 12 IDC-NST samples tested, but do not observe correlations between tumor grade or mitotic index in these samples (data not shown).

We next analyzed a clinical tissue micro array (TMA) cohort of 368 invasive breast cancer samples (Supplementary Table 1) to examine associations between Loxl3 expression and clinicopathological variables. We examined expression of LOXL3 in the cytoplasm and find detectable expression in 76/292 (26.0%) of breast cancer cases (Supplementary Table 2 and Supplementary Fig. 9A). LOXL3 expression is neither prognostic for, nor differentiates between IDC-NST and ILC (Supplementary Table 2; *p* = 0.782). Despite the fact that LOXL3 is specifically expressed by the myoepithelial cells of the mammary gland and the observation that PyMT PDO models show focal upregulation of LOXL3 in CK14 positive cells that are in contact with collagen, we do not find an association between LOXL3 expression and triple negative/basal (ER^NEG^/PR^NEG^/HER2^NEG^) breast cancers (Supplementary Table 2; *p* = 0.721). Also, we do not find associations between tumor size, mitotic index or lymph node status (Supplementary Table 2, *p* = 0.918, *p* = 0.919, *p* = 0.138, respectively). Finally, LOXL3 protein expression does not correlate with overall survival in invasive breast cancer based on the cohort shown in Supplementary Table 1 (Supplementary Fig. 9B).

In short, we find that LOXL3 is upregulated in collectively invading ductal-type breast cancer organoids in cells that are in contact with collagen matrices and at the invasive front in IDC-NST.

## Discussion

Our work shows that Loxl3 expression by basal-type cells at the invasive front of ductal type breast cancer promotes collective invasion. This finding suggests that acquisition of basal features is key to the collective invasive phenotype of luminal breast cancer cells, an assumption that agrees with previously published work by others [14, 42]. Interestingly, propagation of a partial epithelial to mesenchymal transition (EMT) has also been attributed to Loxl2, an enigmatic lysyl oxidase that -like Loxl3- was linked to functions beyond substrate stiffening, such as induction of a Snail-dependent E-cadherin downregulation [20, 43–46]. Because these functions are most likely catalytic independent, it may suggest that Loxl2 plays a more diverse role in tumor biology than Loxl3. Nonetheless, we detect downregulation of E-cadherin-based junctions in the leader cells of the collectively invading strands, suggesting that Loxl3 expression in PyMT cells might also induce a partial and/or temporal EMT during collective invasion. Furthermore, attenuating catalytic activity using the lysyl oxidase family inhibitor βAPN successfully inhibits collective invasion in these basal-type cells, suggesting that ECM remodeling by basal-like luminal leader cells precedes initial collective invasion. Because these cells are low in E-cadherin expression, it is still unclear how modulation of cadherin-based cell-cell contacts contribute to lysyl oxidase-dependent collective invasion in ductal-type breast cancer. However, it is clear that E-cadherin loss of function in the PyMT models results in reduced proliferation and survival [47]. Regardless, systemic increase of stiffness through Threose or Ruthenium-induced crosslinking inhibits collective invasion, while temperature induced bundling promotes an increase in Loxl3 expression and subsequent collective invasion. We, therefore, conclude that collective invasion indicates a stringent requirement for spatiotemporal control over ECM viscoelasticity and stiffness. As such, we hypothesize that collective invasion of ductal breast cancer depends on an instigating pulse of Loxl3 upon local collagen fiber alignment, followed by crosslinking and bundling of collagen fibers. This local stiffening of the ECM will then enable a build-up of mechanical forces, leading to nuclear YAP, actomyosin contraction, and subsequent pro-invasive cell morphology changes [48].

Our data indicate that initiation of these events drives collective invasion through an activation loop as had been proposed for Integrin α5-induced and actin-dependent YAP activation in liver cancer [49]. During this process, Loxl3-induces local stiffening and invasion would coincide with a dynamic, transient, and reversible dismantling of epithelial junctions to propagate collective invasion. In this scenario, Collagen-I-induced transcriptional programs cause local collagen alignment and stiffening, followed by a dynamic control over invasion through RhoA and F-actin-dependent cues, which has also been linked to inhibition of differentiation of progenitor or stem-like cells [50]. In line with this concept, we observe changes in cell morphology, i.e., flattening of the basal-type invading cells against the collagen substratum that is accompanied by a partial loss of luminal epithelial features. Moreover, the fact that nuclear YAP [51] and Loxl3 (this manuscript) are markers of myoepithelial cells in the normal mammary gland, further supports the notion that luminal-type PyMT cells locally trans-differentiate during collective invasion in Collagen-I rich matrices. This temporal and spatial/regional regulation of ECM stiffness by invading tumor cells may be responsible for the lack of correlations between LOXL3, clinicopathological parameters, and breast cancer subtypes in our TMA cohorts. Although we observe LOXL3 expression prominently in approximately half of the whole slide sections analyzed, these LOXL3 enriched focal regions can be easily missed when analyzing 3 mm tumor cores on a TMA. Future studies using larger sets of whole slide tissues will be needed to address this issue.

Interestingly, single cell invasion of mouse lobular breast cancer cells in collagen matrices is not affected by either systemic stiffening of the matrix through induction of crosslinks or inhibition of Lysyl oxidases. Although the expression of Loxl1 was recently linked to invasive lobular breast cancer (ILC) progression in xenograft models [52], the effects appeared mainly due to Loxl1-induced tumor growth. Since the currently available human lobular breast cancer models do not show invasion in 3D Collagen-I matrices (our unpublished findings and [53]), and our data showing that mouse ILC invasion does not depend on LOX activity, it remains unclear if lysyl oxidases produced by cancer cells contribute to stiffening of the local ECM and subsequent single cell dissemination of human ILC.

As mentioned, we find that generic Lox inhibition or systemic ECM stiffening fully inhibits collective invasion of basal-like PyMT cells. Combined with our knockdown and overexpression studies, it, therefore, appears that collective invasion in this model relies on dynamic matrix conditions that provide scale-dependent Loxl3-dependent active stiffening. We propose that structure is dominant over stiffness in the propagation of collective cancer invasion. Our findings fully corroborate recent data showing that collective invasion of breast cancer cells requires matrix remodeling and elevated actomyosin-dependent cellular forces, which cooperatively drive non-linear and scale–dependent matrix stiffening and MMP-induced softening [54]. In contrast, mouse ILC cells migrate through Threose-stiffened Collagen-I gels and invade 3D matrices independent of Lox activity. We envisage that because ILC cells migrate as amoeboid-like single cells and are less dependent on matrix malleability and stiffness, they are less dependent on matrix modifications that promote collectively invading ductal breast cancer cells. These features may explain the characteristic diffuse growth patterns and resulting challenging diagnostic detection of lobular breast cancers.

In conclusion, we find that collectively invading ductal-type breast cancer cells depend on collagen remodeling in part by Loxl3. Transcriptional activation of this factor is enhanced by a Collagen-I rich ECM in luminal cells that undergo a transient and partial EMT. Because this coincides with downregulation of E-cadherin and increased nuclear YAP expression, we think that the current mechanism provides a self-propagating signal for luminal breast cancer cells that have ‘hijacked’ myoepithelial features for collective cancer invasion. These tumor progression features are locally and temporally controlled by distinct micro-environments that are in vivo presented by a plethora in different ECM compositions. As such, a better understanding is required on the detailed identification of specific matrix substrates and the dynamic interplay between the ECM modifying factors produced by the invading tumor cells in response to those invasion niches. Given the specific requirements of collective invasion for spatial and cancer cell-induced ECM stiffness and the differential responses of ductal and lobular breast cancer cells to inhibition of lysyl oxidases, our finding may present options for the future diagnosis or treatment of specific breast cancer sub-types.

## Materials and methods

### Organoid cultures and quantifying invasion

Establishment of the invasive and non-invasive organoid clones from the PyMT mouse model of ductal-type breast cancer [23] will be specified elsewhere. Detailed information on organoid culturing and the accompanying experimental conditions can be found in the Supplementary information.

### Antibodies and reagents

The following antibodies and dilutions were used: F-actin/Phalloidin Alexa Fluor 488, 568 or 647 (1;100, Invitrogen A12379/A12380/A22287), Rabbit anti-YAP (1:200, Cell Signaling #4912), Rabbit anti-Loxl3 (1;200 LSBio LS-C295247), Rat Cyto-Keratin 8/CK8 (1:20, DSHB TROMA-1), Rabbit Cyto-Keratin 14/CK14 (1:200 Biolegend #905301), Mouse anti-Cytokeratin Pan Type I/II (1:500, Thermo Fisher MA5-13156). Secondary antibodies goat anti-Rabbit Alexa Fluor 488/568/647 and goat anti-Rat Alexa Fluor 488/568/647 (1:600, Invitrogen). Collagen was visualized by using either DIC internal reflection within the confocal microscope or GFP-CNA35 probe (see DNA and shRNA section). For Western blot analysis, Rabbit Anti-GFP (FL) (1;500 Santa Cruz sc-8334), and Goat Anti-AKT1 (C-20) (1;1000 Santa Cruz Santa Cruz sc-1618), Alexa Fluor 680 Donkey anti-Goat (1;5000 Thermo Fisher Scientific A-21084), IRDye 680RD Goat anti-Rabbit (1;5000 Li-Cor 926-68071).

### DNA and shRNA constructs

The following DNA constructs were used in this study: GFP-CNA35 (AddGene #61603); pFUTG-blast (described in [55]); pCMV6-LOXL3-Myc-Flag (OriGene, CAT#: MR210480); and pEGFP-N1-ro2GFP (Addgene #82370). PCR based strategies were used to create pFUTG inducible Murine Loxl3 knockdown - (See Supplementary Table 3), and the Murine Loxl3 overexpression vectors.

### Conditional knockdown and transduction

We generated an inducible Loxl3 knockdown vector using an adapted pFUTG-blast as starting vector, as described previously [55]. Viral production, transduction, and selection were done as described [56]. After selection, organoids were treated with doxycycline (2 µg/mL) for 3 days (in BME) or 7 days (in collagen) to verify the knockdown using RT qPCR.

### Confocal imaging on organoids and patient material

Imaging of fixed organoid samples in 3D or patient material was performed on a LSM880 confocal microscope using a 40x water objective (NA 1.1). For YAP localization, z-stacks were taken with 0.75 µm step size. For ILC, single slices were used for YAP analysis. For determining collagen alignment, care was taken to image organoids that were not in the vicinity of other organoids.

### Statistics

Unless stated differently, statistical tests were performed in Microsoft Excel and GraphPad Prism 8 using the, Student’s *T*-test, Mann–Whitney test, or Kruskal–Wallis test for non-Gaussian distributions. Unless stated differently, a minimum of three independent biological replicate experiments were taken into account. Error bars indicates standard error of the mean (SEM) or standard deviation (SD), per figure indicated. Statistical testing on the clinical tissue micro array of invasive breast cancer was performed using IBM SPSS Statistics version 27.0.0.0. Associations between categorial variables were calculated using the Pearson’s Chi-square test. *P*-values <0.05 were considered statistically significant.

### Supplementary methods

Additional experimental procedures including singe cell mRNA sequencing and analysis, collagen probe production, cloning of the Loxl3 lentiviral expression construct, collagen pore size quantifications and alignments, turbidity assays, rheology measurements, immunohistochemistry, and immunofluorescence are available in the Supplementary information.

## Supplementary information

Supplemental_Combined_Single_File
