# Supplementary material for: Spatial collagen stiffening promotes collective breast cancer cell invasion by reinforcing extracellular matrix alignment
Source: Oncogene. 2022 Mar 15;41(17):2458–69. doi: 10.1038/s41388-022-02258-1 (PMC9033577; doi:10.1038/s41388-022-02258-1)
Supplement: Supplementary file 1 — Supplemental_Combined_Single_File [file 41388_2022_2258_MOESM1_ESM.pdf]

Supplemental Materials:

This document contains:

- Supplemental Material and Methods
- Supplemental Figure Legends: 1 - 9
- Supplemental Table Legends: 1 - 3
- Supplemental References
- Supplemental Figures: 1 - 9
- Supplemental Tables: 1 - 3

Supplementary Methods:

Single cell mRNA sequencing and stratification of cell populations

Parental PyMT organoids were cultured in collagen or BME and subjected to single cell mRNA sequencing. Briefly, organoids were cultured in BME, treated with Dispase for 15 minutes and reseeded in collagen type 1 (37 degrees) or BME matrices. After 5 days, collagen and BME samples were treated with collagenase (Cat# C0130, Sigma-Aldrich) for 3mins to disrupt the surrounding ECM and ensure comparability. Organoids were dissociated with trypsin for 3mins and filtered to remove remaining ECM and cell clusters before being resuspended in FACS buffer (0.5mM EDTA and 5% FCS). Single viable cells were sorted into three 384 well plates per condition. Single-cell RNA-sequencing was performed by Single Cell Discoveries (Utrecht, The Netherlands). Briefly, cells with low quality reads, as determined by both high ERCC spike-in percentage and transcript number, or high mitochondrial reads were removed, and mRNA expression data were normalized. Cells with <2000 transcripts and genes with <5 transcripts were excluded from further analysis. Remaining cells were clustered using k-medoids clustering under a Jaccard's similarity criterion of >0.7. To calculate outliers, a negative binominal distribution was assumed and genes in the tail of this distribution at $p<10^{-20}$ were considered outliers and excluded from further analysis. Outlier cells (cells that contained at least two outlier genes) were removed from the analysis. T-SNE maps were computed using a perplexity of 50. Based on these criteria, three clusters were identified in the Collagen and BME analyses. The average expression for each gene was calculated. Cytokeratin (CK)5 and CK14 markers were used to identify the basal population, while luminal clusters were identified using CD14 [1,2]. Fold changes between Collagen and BME were determined using the following formula: ΔFC = Log2 (Collagen basal/Collagen luminal) - Log2 (BME basal/BME luminal). From this, a list of matrisome-related genes upregulated in basal PyMT cells was compiled in which Loxl3 was identified.

Organoid cultures and quantifying invasion

ILC organoid UMC-DL-WEP10 was established from an ILC that developed in the inguinal mammary gland of a *WAPcre;Cdh1^F;Trp53^F* female conditional mouse [3]. Mouse-derived tumor organoid (MDO) models were grown in Basement Membrane Extract (BME, R&D Systems, cat. nr. 3533-005-02) and passaged 1:3-

1:6 using warm trypsin/EDTA (T3924, Sigma). Cold trypsin inhibitor was added in a 1:1 ratio (Sigma-Aldrich, T9003) together with 10mL of cold Ad+ medium (advanced DMEM/F12 (Gibco) supplemented with 1% Pen/Strep (Lonza), 1% HEPES (Gibco), 1% glutamax (Gibco)). Organoids were pelleted by centrifugation and re-plated in BME or Matrigel at 37°C. Hanging drops of 50 µL supplemented with organoids were formed inside 24 well plates for 30 min at 37°C. Growth medium (Ad+ medium supplemented with B-27 supplement (1x) (Gibco, 17504001), N-acetyl-L-cysteine (1.25 mM) (Sigma-Aldrich, A9165), primocin (50 ug/mL) (Invivogen, ant-pm-2) and FGF-basic (42.5 ng/mL, 2.5 nM) (Gibco, 10612074)) was subsequently added until the gels were fully submerged. Medium was refreshed every other day.

For experiments, MDOs were collected from BME or Matrigel using 6 mg/mL Dispase Gibco, 17105041) for 15 mins. At 37°C. Organoids were washed with cold Ad+ medium before usage. Collagen networks were made using Collagen I from rat tail (Corning) at 2 mg/mL at pH 7.0-7.5 (final conditions). In short, collagen was neutralized using 1N NaOH and further diluted using sterilized milliQ water and 10x PBS (1x final concentration). Ad+ medium containing MDOs was added to the NaOH-neutralized solution (1/4 of the total volume). Hanging drops (40 µL) were made directly after neutralization to reduce pre-polymerization on ice [4]. Plates were inverted several times during the first 3-8 minutes to ensure homogeneous MDO distribution within the networks. Collagen gels were polymerized either at 26, 30 or 37°C for a minimal of 45 minutes before 37°C growth medium was added. After this polymerization step, the plate was put in the incubator at 37°C and media were refreshed every other day.

MDO images were taken with an EVOS M5000 microscope using phase contrast. Collectively invading strands were determined by hand in ImageJ after 3 days of culture in collagen. For the Loxl3 knock-down (KD), this was done blinded. The maximum invading length in ILC was determined by fitting an ellipsoid in ImageJ after 2 days of culture in collagen. The major axis was used to determine the maximum invasive length. In some cases, βAPN (Sigma-Aldrich, CDS007521) was added to the medium. βAPN was freshly diluted in medium before use at 0, 10 or 100 µM. Invasion was assessed at day 4 (IDC MDOs) or day 2 (ILC MDOs).

Generation of non-invasive PyMT B6 with Loxl3-ro2GFP overexpression

Loxl3 cDNA (pCMV6-LOXL3-Myc-Flag) was purchased from OriGene (CAT#: MR210480) and pEGFP-N1-ro2GFP was a gift from S. James Remington (Addgene plasmid # 82370; http://n2t.net/addgene:82370 ; RRID:Addgene_82370). pLV-CMV-Loxl3-ro2GFP was generated by multiple insert In-Fusion cloning according to the manufacturer's instructions. PCR-generated inserts of Loxl3 and roGFP2 were captured into the expression vector pLV-CMV-IRES-Puro by recombination between the end sequences of Loxl3 and ro2GFP and AgeI/NheI-linearized pLV-CMV-IRES-Puro vector. PCR primers with a 15-bp overlap that are complementary to the neighboring end were designed with SnapGene: Loxl3-Fw 5'-gacagcgctcaccggtatgagagctgtcagtgtgtggtattgct-3' Loxl3-Rev 5'- agcgctaggacgatctggttactagtctggc- 3' ro2GFP-Fw, 5'- gatcgtcctagcgctaccggactcagat -3' ro2GFP-Rev, 5'-atggcactaggctagcgatcgcttacttgtacagctcgtccatgccgagag -3' (end overlaps in small case). PCR reactions were performed with proofreading containing Phusion High-Fidelity DNA Polymerase (New England Biolabs Inc., #M0530S) using 1 ng template DNA and following the thermocycling conditions for a routine PCR.

Turbidity assays to determine fiber size

Collagen networks were formed inside plastic 1 mL cuvettes for at least 2 hours using DMEM without phenol red (Gibco, cat nr 11039). The optical density was measured using an Ultraspec 2100 pro photo spectrometer between 350 and 900 nm. Samples where collagen was replaced with solvent were used as blank. The turbidity τ was calculated as $\tau = OD\ ln(10)$. Carr's method was used to determine the average mass-length ratio μ and the radius a of the fibers [5], which is valid for randomly oriented, monodisperse rod-like particles that are smaller in radius compared to the wavelength, but longer in length. Fitting was performed using a custom script in Python 2.7. Since there is a large error in the determination of the radius, we focused on the mass-length ratio to compare fiber sizes in different conditions. The average number of monomers per fiber cross-section, N, was calculated using the quarter-staggered molecular packing structure of the fibrils, the molecular mass of a collagen monomer (290 kDa) and the mass-length

ratio: $N=(4.6\ D \cdot \mu)/M$. Here D is the axial (D-banding) periodicity of 67.2 nm.

Quantification of E-cadherin membranous signal

To compare membrane localization of E-cadherin between leader cells and follower cells in the invading strand of PyMT H7 organoids in collagen we determined the Full Width at Half-Maximum (FWHM) and maximum values. We manually drew lines with a width of 10 pixels perpendicular to the membrane, defined by the E-cadherin signal, and plotted line profiles in FIJI. We processed and analyzed acquired line profiles by executing the function *doFWHM* in the "doFWHM.ijm" script for ImageJ by Romain Guiet (https://gist.github.com/romainGuiet/42395587d1193536b7a0858f72db4aca). This script determines Gaussian fits of intensity plots of the line profiles according the function:

$$y = a + (b - a)\exp\left(-\frac{(x-c)^2}{2d^2}\right)$$

and FWHM is solved according:

$$FWHM = 2 * sqrt(2 * ln2) * d.$$

We normalized all maximum intensities (= 'b') by subtracting the baseline value (= 'a') of all Gaussian fits and for visual representation we aligned all peak maxima to x=0. For statistical analysis we removed 3 outliers in the lead cell group as identified by the ROUT analysis routine in Graphpad Prism.

Pore size determination

To determine the pore size of collagen networks in different conditions, we used Ibidi µ slides with 4 wells. These slides were pre-warmed to 26, 30 or 37°C. Collagen networks were formed in these wells and Ad+ medium was added after a minimum of 45 minutes polymerization. Networks were fixed using 4% PFA for 15-30 minutes and stained using GFP-CNA35 overnight. GFP-CNA35 was purified as previously described [6], aliquoted at ~85 mg/mL, snap-frozen and stored at -80°C. It was used at 1:1000 ratio in PBS.

Collagen networks were imaged on a LSM880 confocal microscope using a 60x water objective (NA 1.15). Z-stacks of 10 µm with 0.33 µm steps were used to determine the pore size. To remove noise, a bandpass filter was used with 2-50 pixel thresholds in ImageJ. Stacks were further processed by thresholding using the Otsu method in ImageJ. A custom-made python routine was used to determine the pore size and can be found here: https://github.com/nicojs/quantify-remodeling-imagej-plugin. The routine is based on determining the distribution of the distances between neighboring fibers in the x and y direction [7].

YAP quantification

Confocal imaging was done on fixed IDC organoids in different collagen matrixes and medium conditions. Z-stacks were taken with 0.75 µm step size, and single slices were analyzed for YAP localization. YAP quantification in IDC organoid was performed using a customized program in MATLAB R2018a and will be described in detail elsewhere. Nuclei were automatically detected the based on multi-scale hessian aggregation [8]. Segmentation of the cells was done based on CK8, CK14 or F-actin expression. Cells were automatically characterized as being in contact with the collagen ('outer') or not ('inner'). Cells characterized as 'outer' or 'inner' were visually checked with the z-stack to make sure these cells were in contact with Collagen. Cells that were in contact with the collagen matrix are reported in this manuscript. To determine YAP nuclear translocation in invading cells from ILC organoids, which invade mostly as single cells, we drew regions of interests (ROIs) by hand in ImageJ using the DAPI and F-actin signal on the cells furthest away from the organoid center (*i.e*, the cells leading the invading patterns). Data from at least 3 separate experiments were combined.

CNA35 Probe purification

B21 *E.coli* bacteria expressing recombinant CNA35 probe were pelleted and lysed in 20ml lysis buffer (50mM Tris HCL, 200 mM NaCl, 5 mM Imidazol, and PMSF 0,1mM) and sonicated (amp:100, cycle 0.9) for 7 minutes on ice. Lysed pellet was distributed over high-speed centrifugal tubes and spun down for 1 hour at 20000RPM 4°C. After centrifugation, the supernatant with the CNA35 probe was run over a home-made Nickel purification column at 4°C (cold room) and eluted in 50mM Tris HCL, 200mM NaCl, 300 mM

Imidazol (pH 8.0). Recombinant CNA35 probe purification was verified by running the purified fractions on an SDS-PAGE gel and Coomassie Blue staining. Second step purification was done using a Superdex 200 Hiload 26/60 column. Column was washed with 50mM TRIS HCL, 100mM NaCl ph8.0. Recombinant CNA35 probe purification was verified by running the purified fractions again on an SDS-PAGE gel and Coomassie Blue staining. Fractions that contained the probes were concentrated using centrifugation, and final concentration determined using Bradford. Aliquots were snap frozen in and stored in -80°C. The working concentration of CNA35 is 100µg/ml.

SDS-PAGE and Western blotting

SDS-PAGE and Western blotting analysis was performed as described previously[56] and protein levels were visualized using the Amershan Typhoon Biomolecular Imager.

Rheology

The stiffness of collagen networks was determined using a cone-plate geometry (CP40-2) and a discovery HR2 rheometer (TA Instruments). Collagen networks were allowed to form unperturbed for a minimal of 1 hour at 26°C, 30°C or 37°C in the presence of a liquid trap to prevent evaporation. After network formation, the stiffness was measured at 0.5% strain at 0.5 Hz for 5 minutes to ensure complete polymerization. The temperature was then either increased to 37°C, or kept at the original temperature, and stiffness was followed for an additional 30 minutes. The stiffness reported in S1, S2B and 3B is the stiffness at the end of the additional 30 minutes mark.

Determining collagen alignment

To analyze collagen alignment, we use a publicly-available ImageJ plugin OrientationJ developed by Daniel Sage at the Biomedical Image Group (BIG), EPFL, Switzerland. We analyzed single slices of immunofluorescent images where Collagen was either labelled with GFP-CNA35, or imaged using confocal reflection microscopy. We used OrientationJ to analyze the local orientation (ranges from -90° up to 90°) and coherency (ranges from 0 to 1) of every pixel in the image. To determine the local derivative,

we used a Gaussian gradient with window size of 2 or 3 pixels. Next, a custom-made ImageJ plugin was developed to analyze ~30x150 µm rectangular regions perpendicular to the cell-ECM border to determine Collagen alignment decay over larger distances. The zero distance was set at the cell-matrix interface, where the F-actin or cytokeratin signal determined the cell edge. Using the orientation measurements, *S*, the nematic order parameter is calculated from the second-order tensor order-parameter S_2:

$$S_2 = \begin{bmatrix} \langle cos2\theta \rangle & \langle sin2\theta \rangle \\ \langle sin2\theta \rangle & -\langle cos2\theta \rangle \end{bmatrix}$$

Here, the brackets <.> denote averages over all orientation measurements. Solving the eigenvalue problem for S_2 yields two eigenvalues, which is the scalar order-parameter *S* familiar for liquid crystals in 2D:

$$\lambda_{1,2} = \pm S = \pm\sqrt{\langle cos2\theta \rangle^2 + \langle sin2\theta \rangle^2}$$

This order parameter quantifies the width of the distribution of orientation measurements: It is 0 for a uniform distribution of angles, while it approaches 1 for a sharply-peaked distribution. In practice, we find for isotropic networks values below 0.2. This neumatic order parameter has previously been used in studies of other biological networks like fibrin and F-actin [9,10]. Since the coherency is low for background data, we only take pixels into account where the coherency is bigger than 0.4.

Introducing AGE crosslinks inside collagen fibers

High concentrated rattail collagen (Corning, 9-11 mg/mL) was aliquoted into sterile Eppendorf tubes on ice. L-threose (Sigma) was freshly dissolved in 0.02N acetic acid and added to 6 mg/ml rattail collagen (final concentration) at 10-100mM. Collagen where 0.02N acetic acid were added without L-threose were taken as control. After two weeks incubation, tests were performed to determine stiffening and structure of the networks. For cell experiments, the threose was remove after the 2-week incubation step using centrifugal filter tubes (30kDa cutoff, Amicon® Ultra) at 4°C. Control measurements were performed to

make sure stiffening still occurred and that the collagen structure was not significantly different compared to the acetic acid controls.

Quantitative real-time PCR

Organoids grown in Matrigel or Collagen-I were collected, snap frozen and stored in the -80 until mRNA was extracted. mRNA was extracted using Trizol (Life Technologies) and a cDNA transcription kit (BioRad iScript cDNA Synthesis Kit) was used according to the manufacturers' instructions. Quantitative real-time PCR was performed using Fast Start Universal SYBR Green (Roche) and the BioRad CFX96 Real-Time System. The following primers used: Loxl3-F 5'- CTA CTG CTG CTA CAC TGT CTG T-3' and Loxl3-R 5'- GAC CTT CAT AGG GCT TTC TAG G-3'.

Immunohistochemistry

Immunohistochemistry (IHC) was done on 4µm whole tissue sections diagnosed as high grade invasive ductal carcinoma-no specific type (IDC-NST). The use of material was approved by the Tissue Science Committee of the University Medical Center Utrecht. After antigen retrieval by boiling for 20 minutes in 10 mM citrate pH 6.0 or Tris/EDTA pH 9.0, a cooling period of 30 minutes preceded the primary antibody incubation. IHC procedures were performed standardized on the Roche Ventana™. Hematoxylin was used as a counterstaining. Tissue micro array (TMA) scoring was done blinded to patient characteristics. Scoring of the TMA tumor spots was done by four independent observers using SlideScore. Of each patient, three tumor spots were present on the TMA slide. Protein expression and localization of Loxl3 was scored by positivity for cytoplasmic localization and semi-quantitatively scored as 0 (negative), 1, 2 or 3 (all positive). To minimize the possibility for false-negative/positive scoring, at least four out of a minimal of six tumor spots per patient had to be scored to be considered positive (66%), and >20% of the tumor cells in the core had to express Loxl3.

Immunofluorescence

Formalin fixed paraffin embedded breast tissue sections were deparaffinized, followed by antigen retrieval by boiling in Tris-EDTA buffer for 20 minutes. Sections were washed with 1x PBS and blocked with 1% BSA in PBS for 30 minutes before antibody and DAPI incubations, mounted in ProLong™ Diamond Antifade (Thermo Fisher P36961) and imaged using a Zeiss LSM 880 confocal microscope and 20x objective (NA/0.75).

For immunostaining, MDOs embedded in BME or collagen I were fixed with 4% paraformaldehyde (PFA; Sigma-Aldrich) for 10 minutes at room temperature (20°C) and washed 3 x with 1x PBS. Fixed samples were blocked using 10% normal goat serum (Gibco) in 0.3% Triton-X (Sigma-Aldrich) in 1x PBS for 1h. Primary antibodies were diluted in antibody buffer (0.3% Triton-X with 1% w/v BSA in 1x PBS) and incubated overnight at 4°C with shaking. Next, samples were washed at least 4x with 1x PBS for 10-15 min each and subsequently incubated with secondary Alexa-fluor-488/546/647-conjugated antibodies (1:600), together with DAPI (1 µg/mL), in antibody buffer at 4°C with shaking for at least 16 hours. Samples were imaged using Zeiss LSM 880 using the following objectives (20×/NA 0.75).

Supplemental Figure Legends:

Supplementary Figure 1.

Leader cells in collectively-invading PyMT H7 organoids downregulate junctional E-cadherin. **A**. Immunofluorescence showing expression of E-cadherin (green) and CK14 (purple) in leader (open arrowhead) and follower cells (solid arrowhead) in PyMT H7 organoids in collagen. **B and C**. Quantification of E-cadherin expression. Inverted false-color image of E-cadherin expression from A, used to illustrate positioning of the line scans in leader (grey lines) and follower cells (green lines) perpendicular to the adherens junction (B). Size bar represents 50μm. Gaussian fits of all analyzed E-cadherin line profile signal intensities are shown in (C). Green lines (n=46) were positioned in follower cells (followers) and black lines (n=34) in leader cells (leaders). **D-F**. Quantifications of the full width half maximum (FWHM) values (E) of the lines scans shown in (C), according to the method depicted in (D) and as detailed in Supplemental Materials. Quantifications of the maximum peak intensities (grey values) of the signals measured in (C) are shown in (F). Green violin plots represent follower cells (followers) and white violin plots represent leader cells (leaders). Statistical significance was calculated using Mann-Whitney testing.****p<0.001. Error bars show standard error of the mean.

Supplementary Figure 2.

Quantification of collagen stiffness fully polymerized (control, c), or pre-polymerized for 45 min ('pre', pre-polymerized) matrices at the indicated temperatures. Statistical significance was calculated using Mann-Whitney testing. ***p<0.005. Error bars show standard error of the mean.

Supplementary Figure 3.

The effect of temperature-dependent polymerization and AGE-dependent cross-linking of the collagen network on matrix stiffness and pore size. **A.** Collagen bundles (green) were visualized using confocal microscopy. Size bar indicates 10 μm. **B** and **C**. Quantification of stiffness (B) and pore sizes (C) of the collagen gels shown in (A). Control (0 mM, white bars) and stiffened samples (pre-incubating with threose for two weeks; 100 mM, green bars) are show per polymerization condition. Statistical

significance was assessed by the Mann-Whitney test. *$p<0.05$; **$p<0.01$; ns=non-significant. Experiments were replicated at least 3 times. Error bars show standard error of the mean.

Supplementary Figure 4.

Invasion of ILC cells is not inhibited in response to collagen network stiffening. Mouse ILC MDO cells were seeded in collagen matrices, (pre) polymerized at the indicate temperatures and stiffened using 100µM threose. Cells were visualized using F-actin expression and subsequent confocal microscopy. Red arrows indicate trabecular-type invasion, a characteristic feature of invasive lobular breast cancer. Size bars indicate 50µm.

Supplementary Figure 5.

Light-induced crosslinking reduces collective breast cancer cell invasion. **A.** The principle of the light-induced crosslinking technique. **B and C.** Determining the crosslinking effectivity. Shown is a PAGE gel with the α1 and α2 monomer bands indicated. Collagen dimers and other crosslinked portion is denoted with β. The intensity of the α1 monomer band was normalized to the negative control to show the reduction of the amount of collagen loaded on the gel (C). + = Ruthenium, - = no Ruthenium. **D.** The effect of sodium persulfate ($Na_2S_2O_8$) or Ruthenium (Ru) on cell viability. Viability was determined using an MTT assay. **E.** The effect of blue light on DNA damage. The number of γH2A.x foci per nuclear area was quantified for the different conditions shown. **F and G.** Growth morphologies of the PyMT IDC organoids under different light-induced stiffening conditions in gels that were pre-polymerization at either 37°C or 26°C. Note the reduction of invasion upon light-induced stiffening of the gel (F). Invasion lengths were quantified in triplicate in more than 40 organoids per condition (G). Statistical significance was assessed by the Mann-Whitney test ***$p<0.005$, ****$p<0.001$, ns=non-significant statistical differences. Experiments were replicated at least 3 times. Error bars show standard error of the mean.

Supplementary Figure 6.

LoxL3 is upregulated in the invasive basal-like IDC cells. **A.** PyMT MDO cells were seeded in collagen or BME matrices for 2 days, and the mRNA transcriptomes were defined using single cell sequencing. Matrisome-related genes [11] that were upregulated in collagen in cells expressing basal cytokeratins were identified, and Loxl3 was depicted as candidate follow-up candidate for collagen modulation. LOXL3 has been selected from this list for further investigation. **B - E.** Loxl3 is not sufficient to induce invasion in the non-invasive PyMT organoid B6 in Collagen-I. Western blot showing overexpression of Loxl3 in the non-invasive PyMT organoid B6 (B). Akt was used as loading control. (C) shows a representative DIC image of the organoids cultured for 3 days in Collagen-I gels. Collagen alignment was imaged (white, upper panels) from the organoids shown in (C). Pseudo coloring in the insets indicate orientation angles from -90° to 90° (bottom panels) (D). Size bars represent 50 µm. Collagen alignment (Collagen bundle angle variability perpendicular to the organoid) was quantified in (E) and statistical significance was calculated using a Mann-Whitney test. Perfect alignment with the organoids is defined as 1, and the cut-off for non (random)-alignment is 0.2. ns=non-significant. Experiments are biological replicates and repeated 3 times. **F and G.** LoxL3 is a candidate YAP target gene. Loxl3 sequences from mouse and human gene promotors were analyzed using the Eukaryotic Promoter Database (EPD) [12]. Depicted are the transcription start site (TSS) and the Transcriptional Enhancer factor TEF (also known as TEA Domain family member) (TEAD1/TEAD2/TEAD3/TEAD4) family of transcription factor consensus sites. TEAD transcription factors utilize the activation domain of YAP for transcriptional activation of target genes [13].

Supplementary Figure 7.

Invasion characteristics of the PyMT MDO invasive and non-invasive models used. **A and B.** Organoids were cultured in collagen matrices for 4 days and visualized using the luminal marker cytokeratin (CK)8 (green) and basal marker CK14 (purple) using confocal microscopy (top panels)(A). YAP expression was visualized as a marker mechanosensitive invasion (bottom panels). Note the prominent nuclear-localized Yap expression in the invasive leader cells of the H7 PyMT MDO clone. Scale bars denote 20 µm. Quantification of the nuclear versus cytosolic YAP localization of

the cells in contact with the collagen matrix is shown in (B). Nuclear to cytosolic YAP ratios in the noninvasive B6 cells (green bars) were normalized to 1. ****p<0.001. Nuclear YAP localization is differentially dependent on stiffness and bundling during collective versus single cell invasion. **C and D.** Collagen fiber bundling, but not stiffness induces nuclear YAP expression during collective invasion of PyMT MDO cells. Shown are confocal images (C) depicting organoid architecture and shape using F-actin expression (red), a DNA marker (blue, upper panels), and YAP expression (white; bottom panels). Quantifications of the YAP nuclear/cytoplasmic ratio for cells in contact with the collagen matrix are shown in (D). **E and F.** Collagen fiber stiffness induces nuclear YAP expression during single cell invasion of ILC MDO cells, independent of collagen bundling. Shown are confocal images (E) depicting organoid architecture and shape using F-actin expression and DNA stainings (upper panels), and YAP expression (white; bottom panels). Quantification of the YAP nuclear/cytoplasmic ratio for cells in contact with the collagen matrix are shown in (F). Size bars indicate 10µm. *p<0.05; ***p<0.005; ****p<0.001. Statistical significance was assessed using the Student *t*-test. Experiments were replicated at least 3 times. Error bars show standard error of the mean.

Supplementary Figure 8.

Inhibition of Lysyl oxidases does not reduce single cell invasion of lobular breast cancer cells. **A and B.** βAPN, a generic Lox family inhibitor, does not inhibit single cell invasion in collagen in UMC-DL-WEP10, a mouse ILC organoid. Size bar represents 30µm. Invasive strand length was quantified and normalized to the 0 µM control in (B). Statistical significance was assessed using the Kruskal-Wallis test, ns=non-significant statistical differences. Experiments are biological replicates and repeated at least 3 times. Error bars show standard error of the mean.

Supplementary Figure 9.

LOXL3 protein expression and Breast Cancer survival correlations. **A.** Shown are two representative tissue micro array (TMA) cores that were scored negative (top panel) or positive (brown color; bottom panel) for Loxl3 protein expression using immunohistochemistry (IHC). Cores were counterstained using

hematoxylin. Size bar is 100 µm. **B.** Kaplan–Meier survival curves according to LOXL3 protein expression status (n = 368). P value was calculates using a Mantel Cox Log Rank test ($\chi 2$ .391; df=1).

Supplementary table legends:

Supplemental table 1: Clinicopathological characteristics of the invasive Breast Cancer TMA cohort.

Supplemental table 2: Correlation of LOXL3 protein expression with clinicopathological features.

Supplemental table 3: shRNA Loxl3 targeting sequences.

Supplemental References

1 Stingl J, Eirew P, Ricketson I, Shackleton M, Vaillant F, Choi D *et al.* Purification and unique properties of mammary epithelial stem cells. *Nature* 2006; 439: 993–997.

2 Li CM-C, Shapiro H, Tsiobikas C, Selfors LM, Chen H, Rosenbluth J *et al.* Aging-Associated Alterations in Mammary Epithelia and Stroma Revealed by Single-Cell RNA Sequencing. *Cell Reports* 2020; 33: 108566.

3 Derksen PWB, Braumuller TM, Burg E van der, Hornsveld M, Mesman E, Wesseling J *et al.* Mammary-specific inactivation of E-cadherin and p53 impairs functional gland development and leads to pleomorphic invasive lobular carcinoma in mice. *Dis Model Mech* 2011; 4: 347–358.

4 Raub CB, Suresh V, Krasieva T, Lyubovitsky J, Mih JD, Putnam AJ *et al.* Noninvasive Assessment of Collagen Gel Microstructure and Mechanics Using Multiphoton Microscopy. *Biophys J* 2007; 92: 2212–2222.

5 Carr ME, Hermans J. Size and Density of Fibrin Fibers from Turbidity. *Macromolecules* 1978; 11: 46–50.

6 Aper SJA, Spreeuwel ACC van, Turnhout MC van, Linden AJ van der, Pieters PA, Zon NLL van der *et al.* Colorful protein-based fluorescent probes for collagen imaging. *PLoS ONE* 2014; 9: e114983.

7 Kaufman LJ, Brangwynne CP, Kasza KE, Filippidi E, Gordon VD, Deisboeck TS *et al.* Glioma Expansion in Collagen I Matrices: Analyzing Collagen Concentration-Dependent Growth and Motility Patterns. *Biophys J* 2005; 89: 635–650.

8 Lou X, Koethe U, Wittbrodt J, Hamprecht FA. Learning to Segment Dense Cell Nuclei with Shape Prior. *2012 Ieee Conf Comput Vis Pattern Recognit* 2012; 1: 1012–1018.

9 Jansen KA, Bacabac RG, Piechocka IK, Koenderink GH. Cells Actively Stiffen Fibrin Networks by Generating Contractile Stress. *Biophys J* 2013; 105: 2240–2251.

10 Alvarado J, Mulder BM, Koenderink GH. Alignment of nematic and bundled semiflexible polymers in cell-sized confinement. *Soft Matter* 2013; 10: 2354–2364.

11 Hynes RO, Naba A. Overview of the Matrisome—An Inventory of Extracellular Matrix Constituents and Functions. *Csh Perspect Biol* 2012; **4**: a004903.

12 Dreos R, Ambrosini G, Groux R, Cavin Périer R, Bucher P. The eukaryotic promoter database in its 30th year: focus on non-vertebrate organisms. *Nucleic Acids Res* 2017; **45**: D51–D55.

13 Vassilev A, Kaneko KJ, Shu H, Zhao Y, DePamphilis ML. TEAD/TEF transcription factors utilize the activation domain of YAP65, a Src/Yes-associated protein localized in the cytoplasm. *Genes & Development* 2001; **15**: 1229–1241.

Supplemental Figure 1

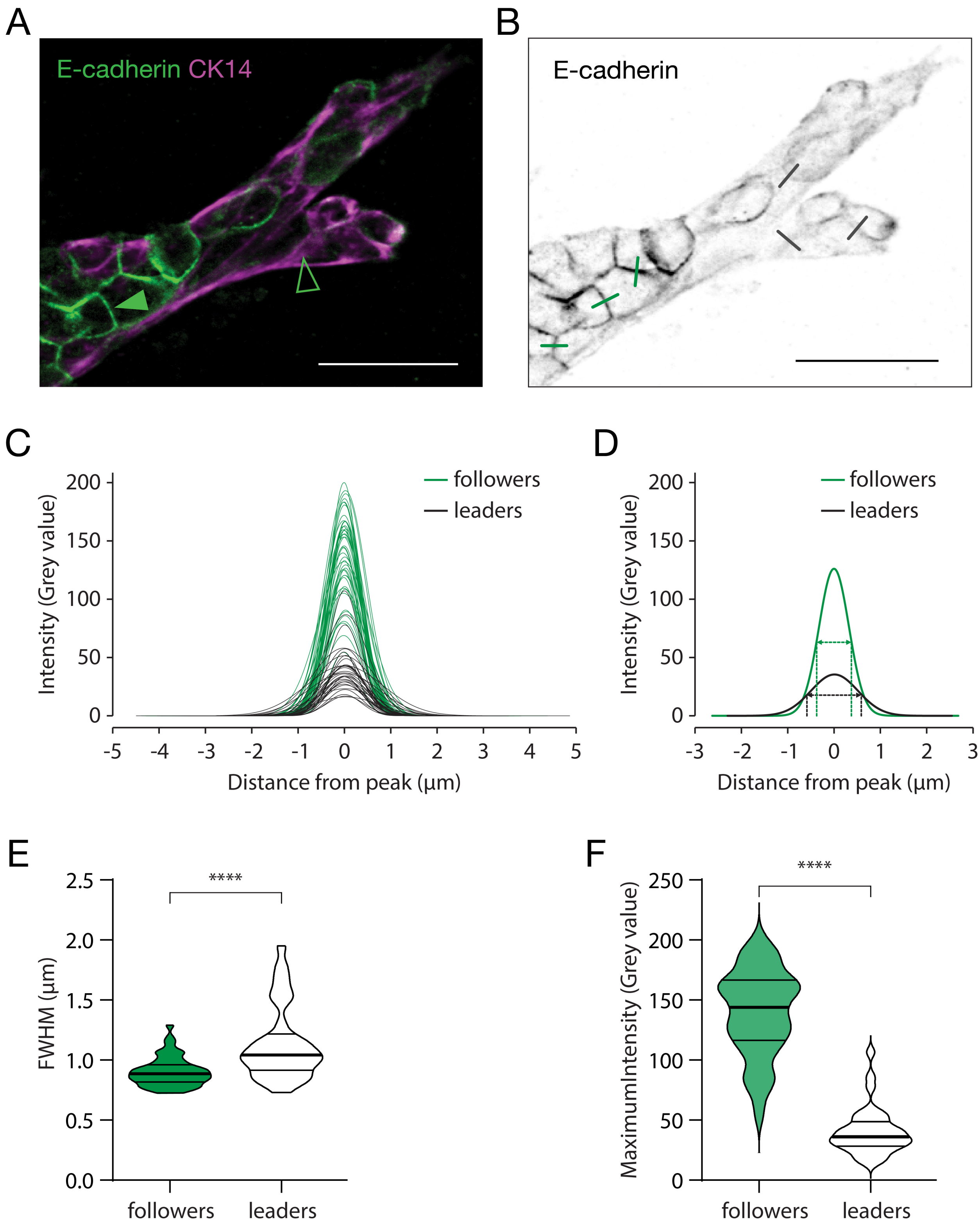

Supplemental Figure 2

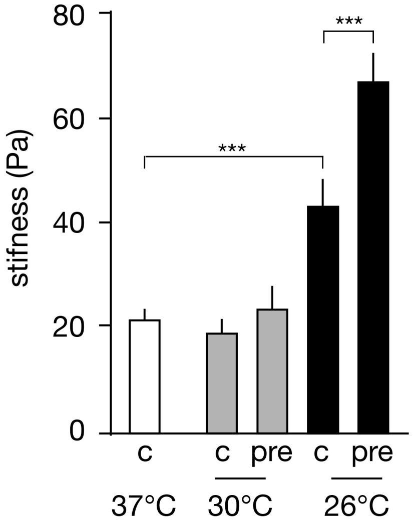

Supplemental Figure 3

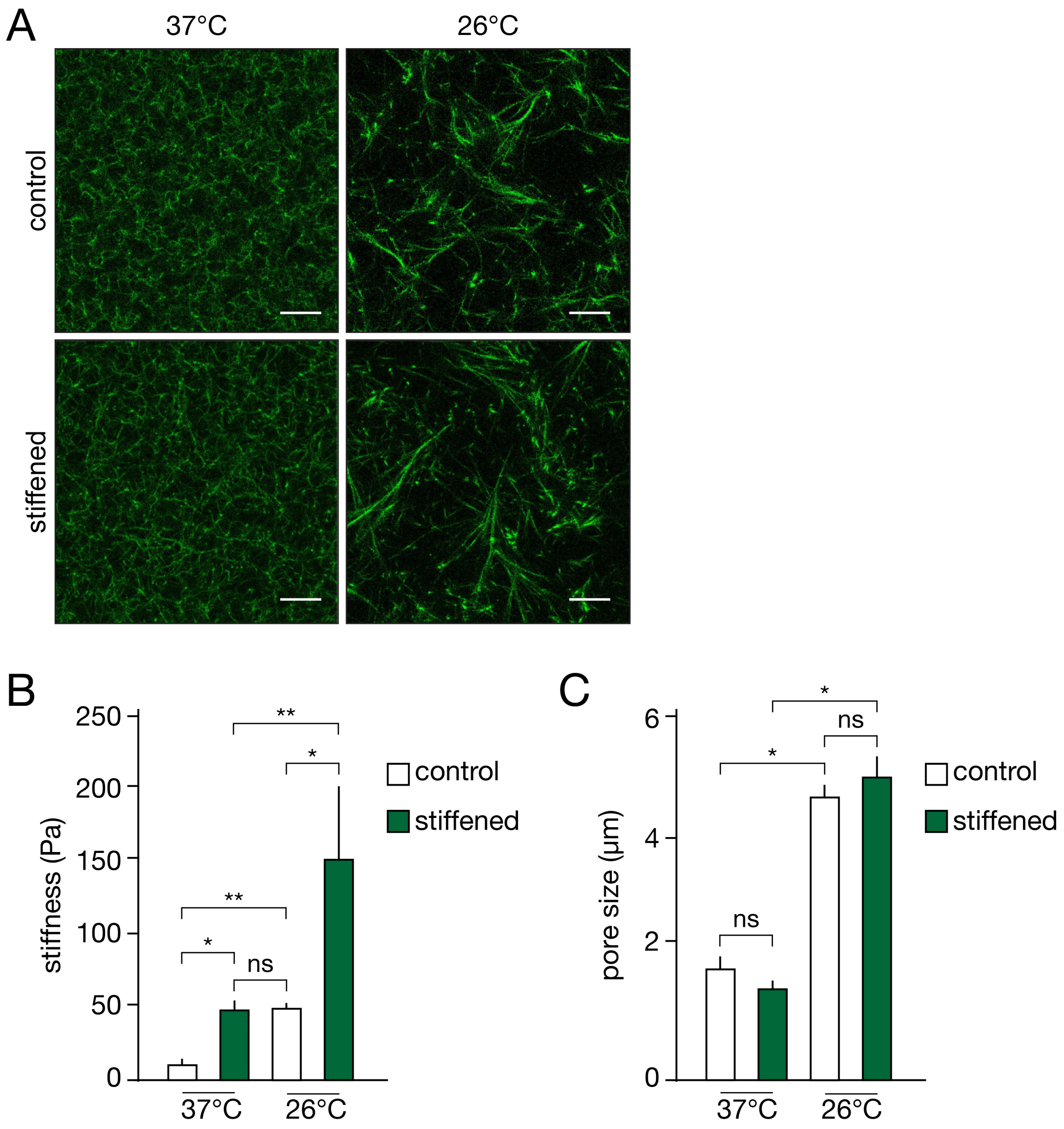

Supplemental Figure 4

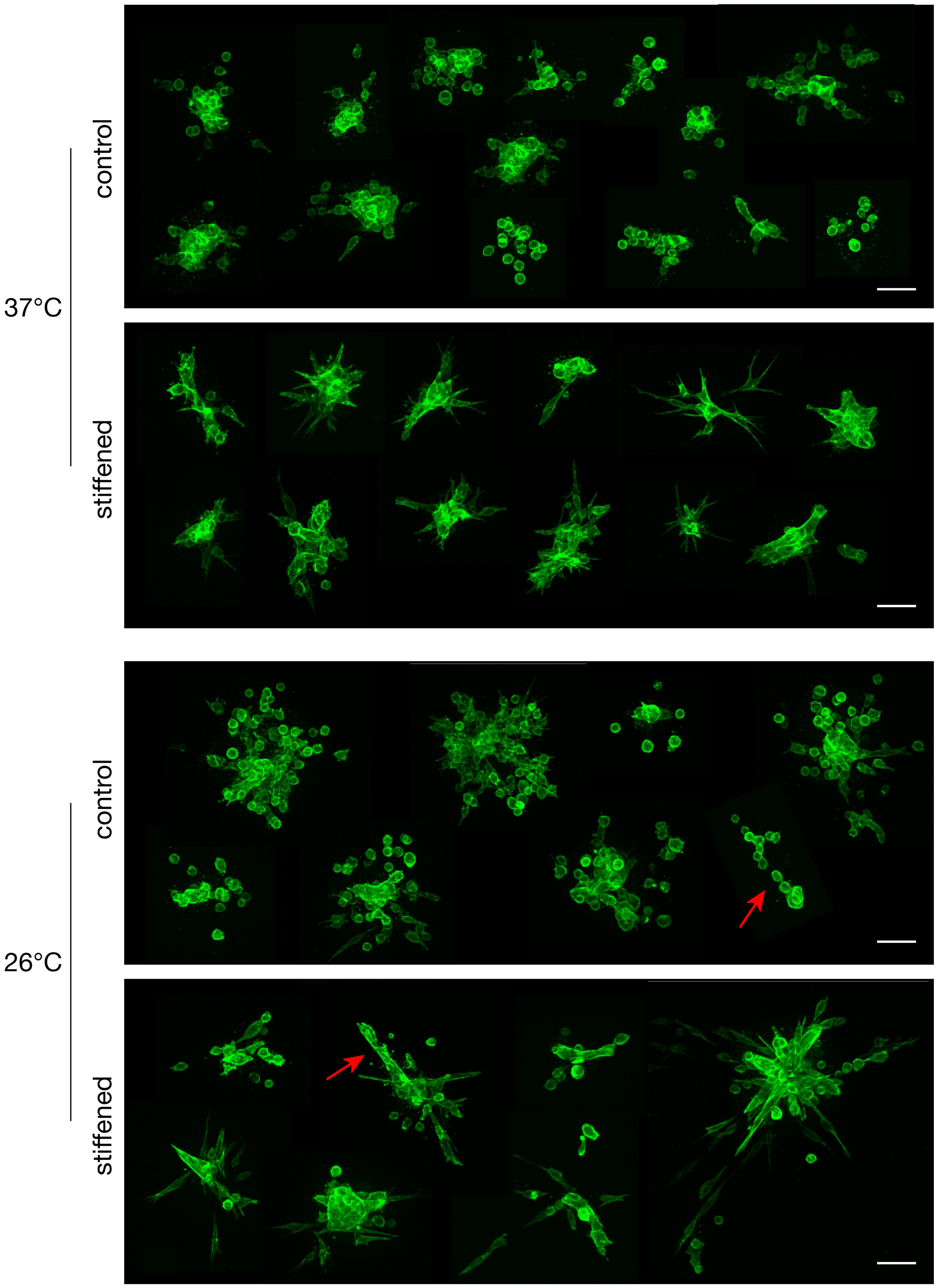

Supplemental Figure 5

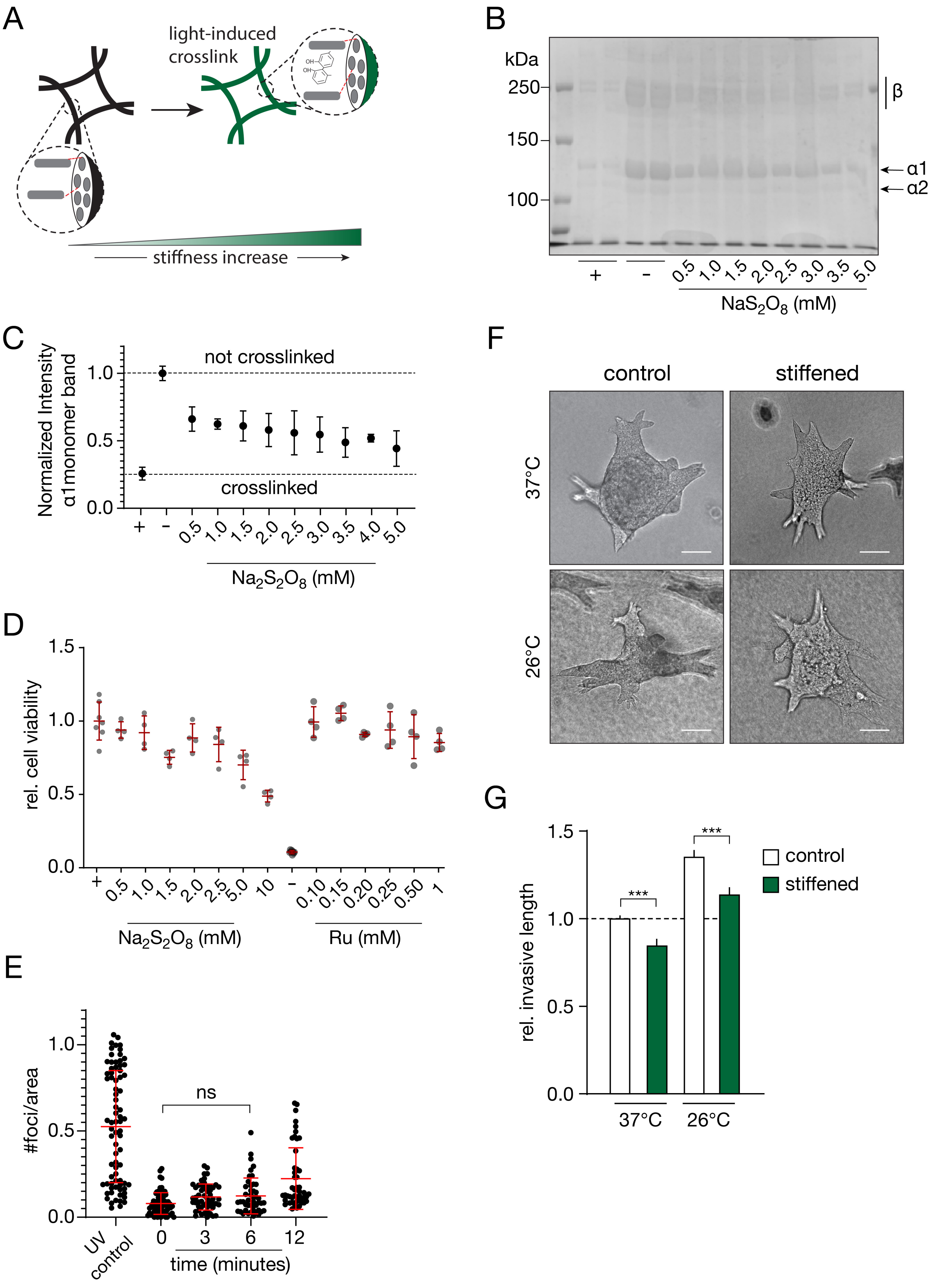

Supplemental Figure 6

A

Matrisome Category	***Gene ID* (Log2FC)**				
ECM glycoprotein	*Tnc* (2.87)	*Tgfbi* (2.02)	*Ecm1* (1.39)		
Collagens	*Lamb3* (1.08)	*Ltbp4* (1.01)	*Col8a1* (2.48)	*Col18a1* (1.19)	*Col7a1* (1.03)
Proteoglycans	*Hspg2* (1.02)				
ECM Regulators	*Adamts1* (1.58)	*Plau* (1.47)	*Loxl3* (1.46)	*Serpinb5* (1.13)	*Adamtsl4* (1.01)
Secreted Factors	*Inhba* (2.53)				

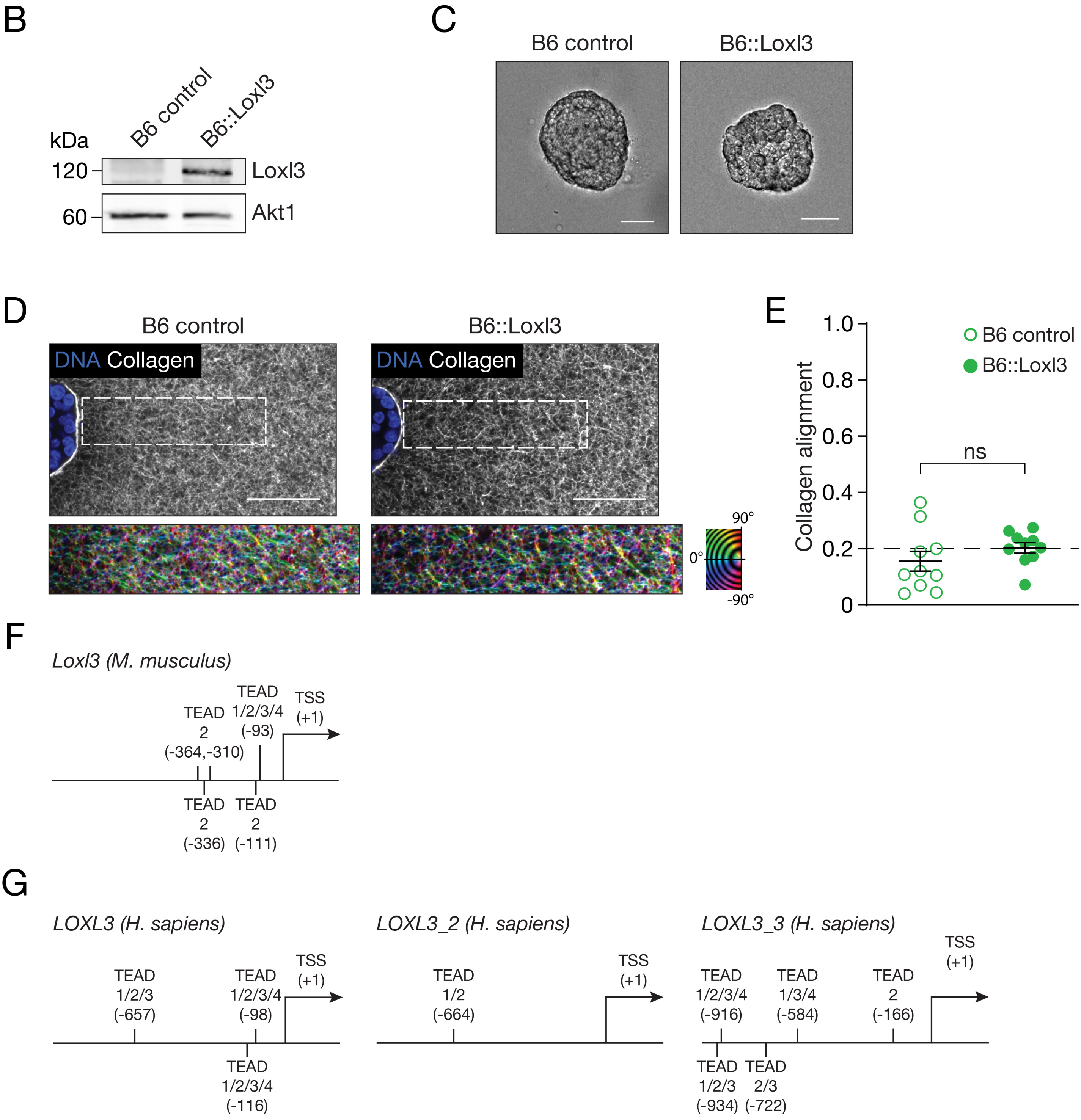

Supplemental Figure 7

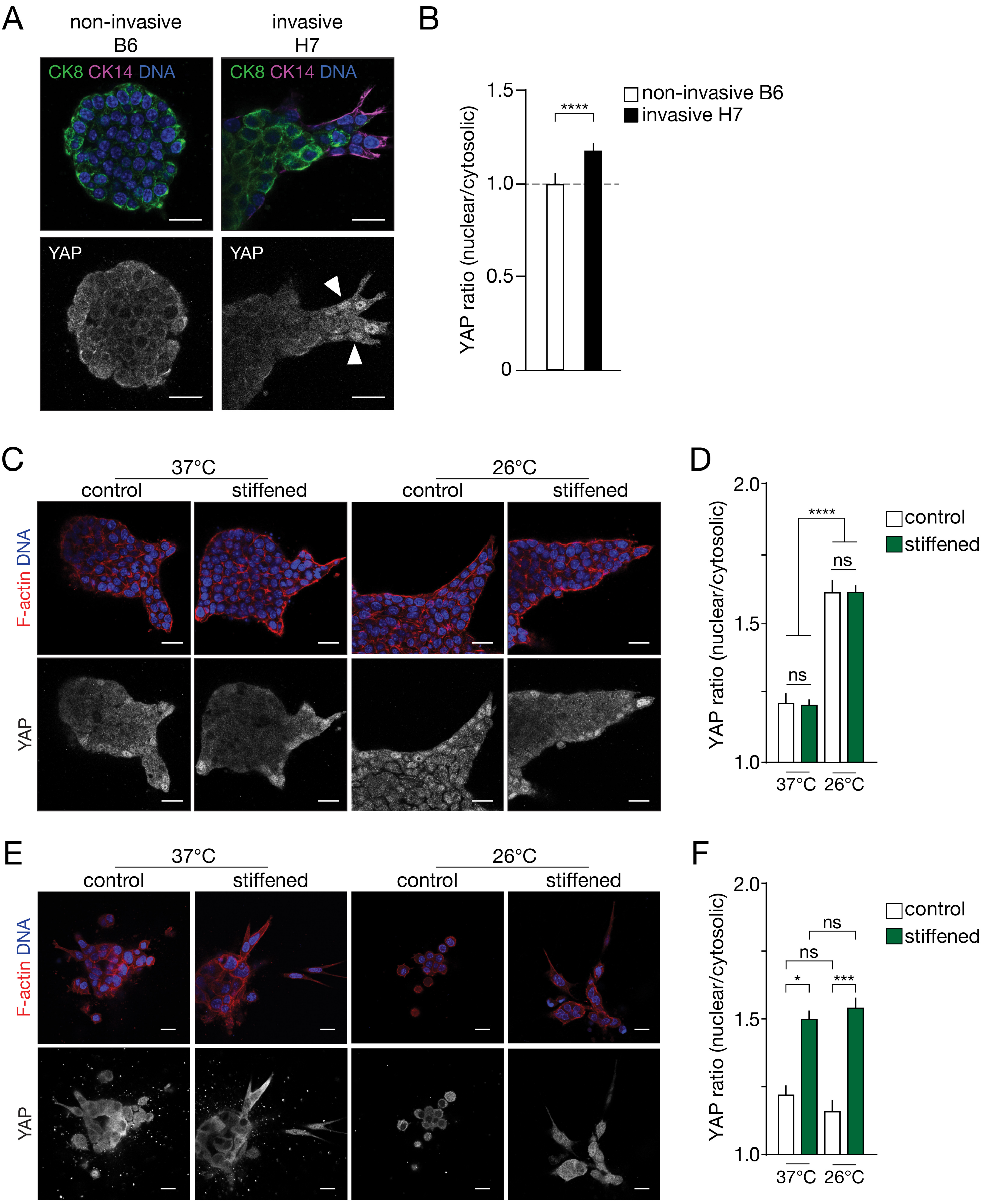

Supplemental Figure 8

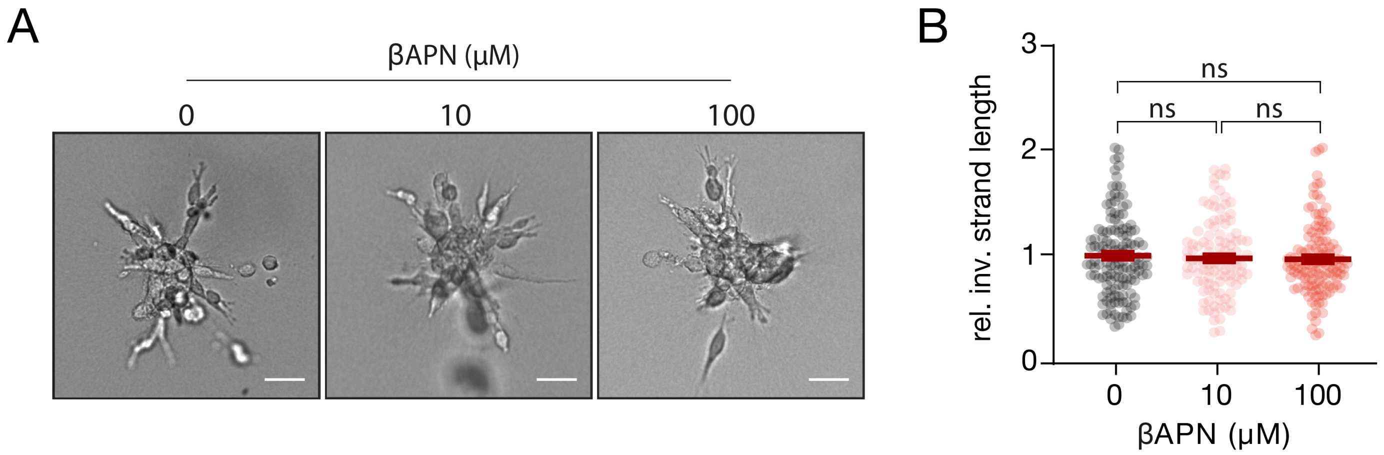

Supplemental Figure 9

A

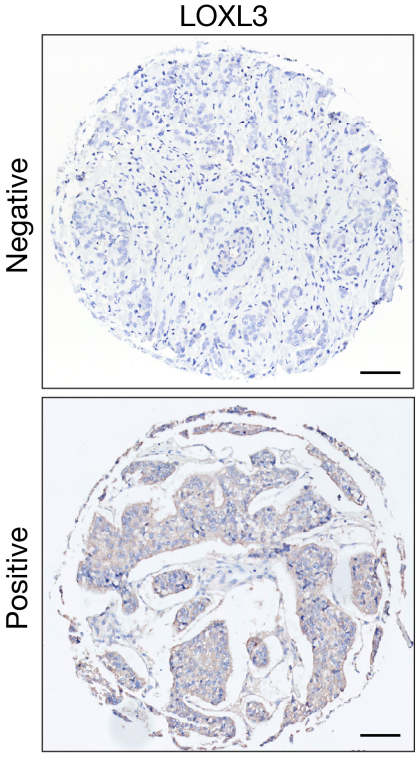

B

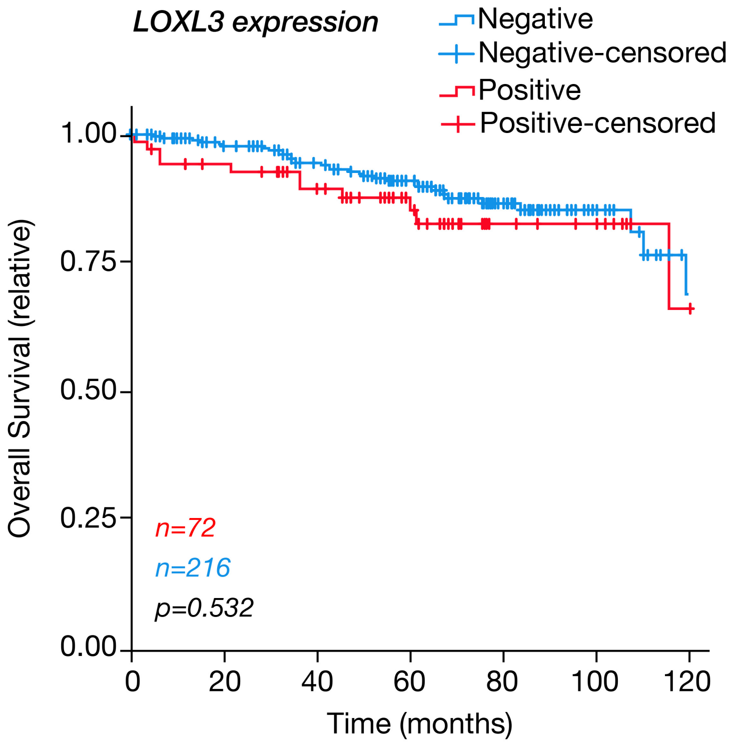

Supplementary Table 1.
Clinicopathological characteristics of the invasive Breast Cancer TMA cohort.

	**Grouping**	**Nr or value**	**%**
Age (years)	Mean	60	
	Range	29 - 97	
Histologic type	IDC	252	68.5
	ILC	39	10.6
	IDLC	62	16.8
	Other	15	4.1
Tumor size (cm)	≤2	183	49.7
	>2 and ≤5	153	41.6
	>5	27	7.3
	Not available	2	0.5
Histologic grade	1	63	17.1
	2	132	35.9
	3	129	35.1
	Not available	44	12.0
MAI (per $2mm^2$)	≤12	208	56.5
	≥13	150	40.8
	Not available	10	2.7
Lymph node status	Negative	134	36.4
	Positive	225	61.1
	Not available	9	2.4
ER status	Negative	53	14.4
	Positive	304	82.6
	Not available	11	3.0
PR status	Negative	116	31.5
	Positive	238	64.7
	Not available	14	3.8
HER2 status	Negative	292	79.3
	Positive	33	9.0
	Not available	43	11.7
Total Patient #		368	100.0

IDC: invasive ductal carcinoma, ILC: invasive lobular carcinoma,
IDLC: invasive ductolobular carcinoma, MAI: Mitotic Activity Index,
ER: estrogen receptor, PR: progesterone receptor
HER2; human epidermal growth factor receptor 2

Supplementary Table 2.
Correlation of LOXL3 protein expression with clinicopathological features.

Clinical Feature	Total	Negative	%	Positive	%	*P-value*
Histological type						
IDC	252	202	80.2	50.0	19.8	
ILC	39	32	82.1	7.0	17.9	
IDLC	62	47	75.8	15.0	24.2	
Other	15	11	73.3	4.0	26.7	
						.782
Histologic grade						
1	64	55	85.9	9.0	14.1	
2	132	99	75.0	33.0	25.0	
3	129	104	80.6	25.0	19.4	
						.187
Tumor size (cm)						
≤2	188	149	79.3	39.0	20.7	
>2 and ≤5	150	120	80.0	30.0	20.0	
>5	30	23	76.7	7.0	23.3	
						.918
MAI (per $2mm^2$)						
≤12	209	166	79.4	43.0	20.6	
≥13	149	119	79.9	30.0	20.1	
						.919
Lymph node status						
Negative	284	111	39.1	173.0	60.9	
Positive	74	22	29.7	52.0	70.3	
						.138
Tripe negative						
ER-/PR-/HER2-	71	67	94.4	4.0	5.6	
						.721

Supplementary Table 3. shRNA Loxl3 targeting sequences.

name shRNA	Sequence	Direction
shRNA1_fw	tgatagagatACCGGCCGCGTTACCTCTAGCCATTTCTCGAGAAATGGCTAGAGGTAACGCGGTTTTTtcgagacgcgtccta	Forward
shRNA1_rev	taggacgcgtctcgaAAAAAGGTCGTTATCAACCCAAATTTCTCGAGAAATTTGGGTTGATAACGACCCCGGTatctctatca	Reverse
shRNA2_fw	tgatagagatACCGGCTAGTAACCAGATCGTCTAAACTCGAGTTTAGACGATCTGGTTACTAGTTTTTtcgagacgcgtccta	Forward
shRNA2_rev	taggacgcgtctcgaAAAAACTAGTAACCAGATCGTCTAAACTCGAGTTTAGACGATCTGGTTACTAGCCGGTatctctatca	Reverse
